# Effect of Initiator Density, Catalyst Concentration, and Surface Curvature on the Uniformity of Polymers Grafted from Spherical Nanoparticles

**DOI:** 10.1021/acs.macromol.5c02737

**Published:** 2026-01-06

**Authors:** Rongguan Yin, Hanshu Wu, Xiaolei Hu, Khidong Kim, Francesca Lorandi, Dagmar R. D’hooge, Edmondo M. Benetti, Michael R. Bockstaller, Krzysztof Matyjaszewski

**Affiliations:** † Department of Chemistry, 6612 Carnegie Mellon University, Pittsburgh, Pennsylvania 15213, United States; ‡ Laboratory for Macromolecular and Organic Chemistry, Department of Chemical Sciences, University of Padova, Padova 35131, Italy; § Laboratory for Chemical Technology, Department of Materials, Textiles and Chemical Engineering, Ghent University, Technologiepark 125, Zwijnaarde, Ghent B-9052, Belgium; ∥ Centre for Textile Science and Engineering, Department of Materials, Textiles and Chemical Engineering, Ghent University, Technologiepark 125, Zwijnaarde, Ghent B-9052, Belgium; ⊥ Department of Materials Science and Engineering, Carnegie Mellon University, Pittsburgh, Pennsylvania 15213, United States

## Abstract

Polymer-grafted nanoparticles (PGNPs) are versatile hybrid materials whose properties critically depend on brush dimensions, uniformity, and grafting density. Herein, we systematically investigated how initiator density, catalyst concentration, and nanoparticle curvature govern the growth of poly­(methyl methacrylate) (PMMA) brushes grafted from spherical SiO_2_ nanoparticles via surface-initiated activators regenerated by electron transfer atom transfer radical polymerization (SI-ARGET ATRP). By tuning the initiator density through a combination of “active” and “dummy” silane initiators anchored on the nanoparticles’ surface and controlling the catalyst concentration, we reveal that increased initiator crowding and smaller surface curvature amplify steric hindrance, leading to decreased initiation efficiency and broader molecular weight distributions. Correlation with the corresponding unattached chains by ARGET ATRP suggests the presence of permanently inaccessible (“buried”) initiation sites, which are a characteristic of surface-grafted systems. At sufficient Cu catalyst concentrations, uniform brush growth is attained across different initiator densities, whereas decreased catalyst concentrations accentuate nonconcurrent initiation and propagation. These findings provide mechanistic insights into the interplay of initiator density, catalyst concentration, and surface curvature, offering design principles for tailoring the PGNP architecture. These results can guide the structural engineering of densely grafted surfaces, including nanoparticles and flat substrates, for applications in nanocomposites, photonics, and functional coatings.

## Graphical abstract

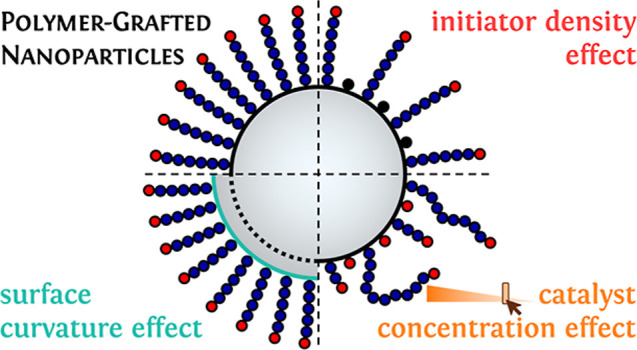

## Introduction

Polymer-grafted nanoparticles (PGNPs), also known as nanoparticle brushes, in which nanoparticles are densely grafted with polymer “canopies” via surface-initiated polymerizations, are regarded as versatile platforms for designing hybrid nanomaterials whose properties surpass those of classical polymer nanocomposites.^1–9^ Chains with precisely controlled initiator density, molecular weight (and distribution), predetermined (co)­polymer compositions, and macromolecular architecture can be grown from nanoscopic cores. This converts the poorly defined particle–matrix interface in classical “particle-in-polymer dispersions” into a chemically programmable domain, allowing the tailoring of interparticle interactions, dispersion, and nanoassembly.^10–14^ Such a level of control enables the direct assembly of brush-modified nanoparticles into one-component hybrid materials with versatile property enhancements, such as mechanical toughening,^15–17^ novel optical responses,^18–20^ electrochemical stability,^21–23^ and biofunctionality,^24–26^ features that are difficult to realize through rule-of-mixture approaches.

Recent advances in nanoparticle surface modification and related polymer grafting methods have expanded the library of accessible PGNP architecture and assemblies.^27–34^ For the “grafting-from” approach, surface-initiated atom transfer radical polymerization (SI-ATRP) has become preferential due to the high grafting density, preserved functionality of polymer chain ends, and compatibility with various functional groups and monomers.^35–38^ For PGNPs, grafting density (σ, chains nm^–2^) represents a pivotal parameter defining the number of polymer chains grafted per unit surface area.^39,40^ While high grafting density (σ > 0.3 chains nm^–2^) endows PGNPs with the ability to self-assemble into uniform and ordered structures,^41^ PGNPs with low grafting density (σ < 0.1 chains nm^–2^) exhibit segregation-driven interactions, which are exclusive for nanomaterials that require immobilized or oriented particles.^11^ Notably, the grafting density is tunable with the assistance of SI-ATRP: First, the capability of nanoparticles to graft polymer chains can be regulated by tuning the relative content of “active” and “dummy” ATRP initiators at the surface (i.e., surface modification reagents with and without reactive C–Br bonds, respectively).^42,43^ Second, by reducing the amount of Cu-based catalyst during polymerization, intentional nonconcurrent initiation and nonuniform chain growth can lead to a lower grafting density.^44^

A previous computational study revealed that the curvature effect of surfaces also influences the grafted polymers (in the following, the term “curvature” will be used as a synonym for the “principal curvature” of the surface of a sphere defined as 1/*R* where *R* is the particle radius).^45^ During grafting from the surface, the crowdedness among the chains hinders reactivation of a significant fraction of chain-ends, thereby causing these chains to remain permanently dormant (or, in a whimsical saying, “buried alive”). These “hindered dormant” chains, in contrast to “dead terminated” grafts (due to radical termination by combination or disproportionation), result in a relatively broader molecular weight distribution of the entire grafted brush layer. Moreover, as the radius of nanoparticles increases (i.e., decreasing surface curvature), this effect becomes more evident, with a larger amount of hindered dormant chains resulting in a broader chain length distribution.^45^ Experimentally, it is challenging to reproduce the predicted formation of hindered dormant chains using SI-ATRP with uniform chain growth, especially when applied to larger nanoparticles (considering the overall reaction system’s viscosity) and a small fraction of formed polymers available for size-exclusion chromatography (SEC) analysis. Being able to distinguish these hindered dormant chains would be of considerable significance for validating theoretical predictions and refining the mechanistic understanding. To the best of our knowledge, such an investigation has not yet been completed.

Herein, we systematically investigated the effect of the initiator density, catalyst concentration, and surface curvature on the growth of polymer brushes grafted from (spherical) nanoparticles. Despite the difficulty of deconvoluting hindered dormant chains in the molecular weight (distribution) curves, such an effect can be detected by forcing a nonuniform chain growth through a gradual decrease of Cu catalyst concentration. Three representative silica nanoparticles (SiO_2_ NPs) with distinct diameters (*d*) were studied: *d*_small_ ≈ 15 nm, *d*_medium_ ≈ 75 nm, and *d*_large_ ≈ 110 nm. The initiator density was adjusted by applying different relative concentrations of “active” and “dummy” initiators for nanoparticle surface modification, while the actual grafting density was regulated by tuning the initial Cu catalyst content.

Surface grafting of poly­(methyl methacrylate) (PMMA) brushes via surface-initiated activators regenerated by electron transfer (SI-ARGET) ATRP revealed that both a higher initiator density and a larger nanoparticle’s *d* increased chain crowding, thereby amplifying steric hindrance among grafted chains. This effect was reflected in a broader molecular weight distribution of the brush layers and a reduced initiation efficiency. Comparison with separately synthesized unattached polymers using a small-molecule initiator further confirmed the presence of permanently buried initiation sites. These findings advance the mechanistic understanding of polymer-grafted nanoparticles and provide valuable guidance for the structural and architectural design of densely polymer-grafted nanoparticles and flat surfaces.

## Results and Discussion

### Silica (SiO_2_) Surface Modifications and Initiator Density Regulation

Three representative colloidal SiO_2_ NP solutions with distinct diameters were used in this study (see the Experimental Section in the Supporting Information). Extensive prior studies have focused on the smallest SiO_2_ NPs (*d* ≈ 15.8 nm), whose surface functionalization involved tethering an excess of 3-(chlorodimethylsilyl)­propyl α-bromoisobutyrate (Scheme S1 and Figure S1) as “active” ATRP initiators in methyl isobutyl ketone.^46–48^ Following surface modification and purification, three model SI-ARGET ATRP reactions were conducted, grafting PMMA from these nanoparticles under conditions with 200 ppm of Cu catalyst relative to MMA, a level sufficient to achieve well-controlled particle brushes. The model “surface bromide density” of the resulting SiO_2_-*g*-PMMA was calculated (eqs 1 and S1–S4). This value, referred to as the initiator density (σ_in_), was estimated to be approximately 0.7 Br nm^–2^.

1 $$\sigma =\frac{\left(1-f_{inorg}\right)N_{A}\rho_{SiO_{2}}d_{SiO_{2}}}{6f_{inorg}M_{n}}$$

where *f*_inorg_ denotes the inorganic fraction of SiO_2_-*g*-PMMA measured by thermogravimetric analysis (TGA) in air; *N*_A_ is Avogadro’s constant; ρ is the density of SiO_2_; *d* indicates the SiO_2_ core diameter; and *M*_n_ is the number-average molecular weight of the grafted PMMA, determined by gel permeation chromatography (GPC).

The spacing between surface-bound initiators was further regulated by anchoring a mixture of “active” and “dummy” silane initiators (Figure 1a).^35^ The “dummy” initiator, chlorodimethylsilane, serves only to occupy surface sites without the capability of initiating polymerization. By systematically varying the molar ratio of these two species, σ_in_ was scaled proportionally, yielding values of approximately 0.4, 0.1, and 0.03 Br nm^–2^. This result aligns with expectations, as each silane reagent is monofunctional, allowing the direct control of the initiator spacing through stoichiometry.

**Figure 1:**
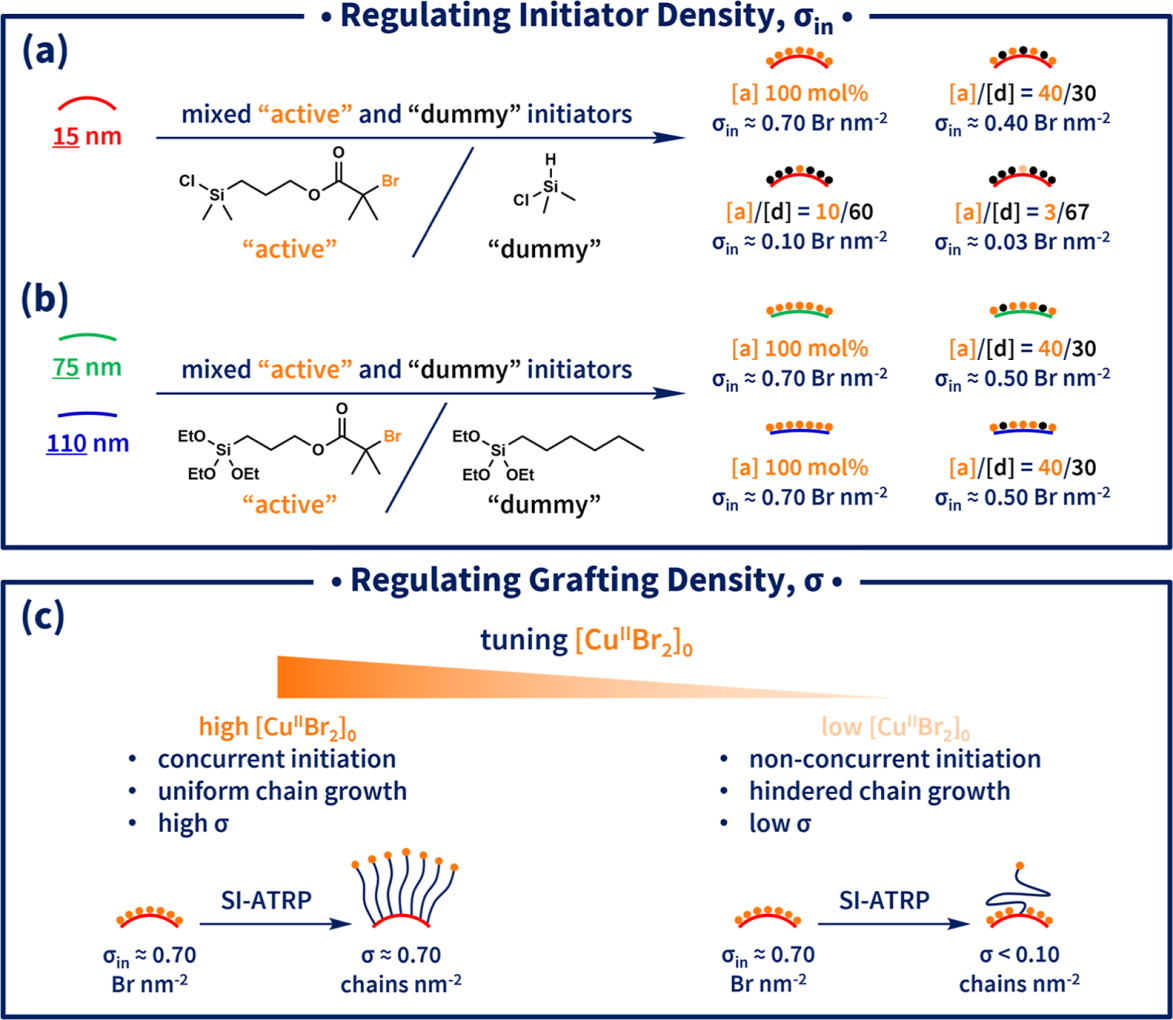
Schematic illustration of regulating (a,b) initiator density (σ_in_, Br nm^–2^) and (c) grafting density (σ, chains nm^–2^) in polymer-grafted nanoparticles.

For the larger SiO_2_ NPs (*d* ≈ 75 and 110 nm), a different surface modification strategy was employed to account for their distinct sample dispersion environments (Figure 1b). Since chlorosilanes readily react with alcohols, 3-(triethoxysilyl)­propyl α-bromoisobutyrate (Figure S2) was selected as the active initiator, while triethoxyhexylsilane served as the dummy initiator for tuning initiator spacing. The solvents (methyl ethyl ketone for 75 nm and isopropyl alcohol for 110 nm, respectively) of the bare SiO_2_ dispersions were completely exchanged into ethanol, a protic medium that facilitates efficient hydrolysis and condensation reactions. Subsequently, (mixed) silane reagents were anchored to the nanoparticle surfaces under basic conditions (see the Supporting Information for details). The modified particles were then purified by repeated high-speed centrifugation (12,000 rpm, 30 min) to remove unbound initiators and dried under ambient conditions, yielding SiO_2_–Br macroinitiators for SI-ARGET ATRP studies.

To accurately quantify the σ_in_ for the larger SiO_2_–Br NPs, the core diameters of SiO_2_-*g*-PMMA after model SI-ARGET ATRP reactions were measured by transmission electron microscopy (TEM) using a random sampling approach (Figure S3, Table S1 for 75 nm, and Figure S4, Table S2 for 110 nm, respectively). Representative TEM images revealed that most of the SiO_2_ cores retained a spherical morphology, regardless of their size (Figure 2). However, the 75 nm SiO_2_ NPs displayed a broader size distribution and some core aggregations prior to surface modification, compared to the more uniform 15 and 110 nm samples. The average core diameters were determined to be 73.3 ± 19.6 and 111.4 ± 5.0 nm for the two larger particle types, and these values were used for estimating σ_in_. As the 15 and 110 nm SiO_2_ NPs exhibited relatively narrow size distributions, subsequent comparisons of the resulting polymer brush layers were expected to yield empirically grounded trends.

**Figure 2:**
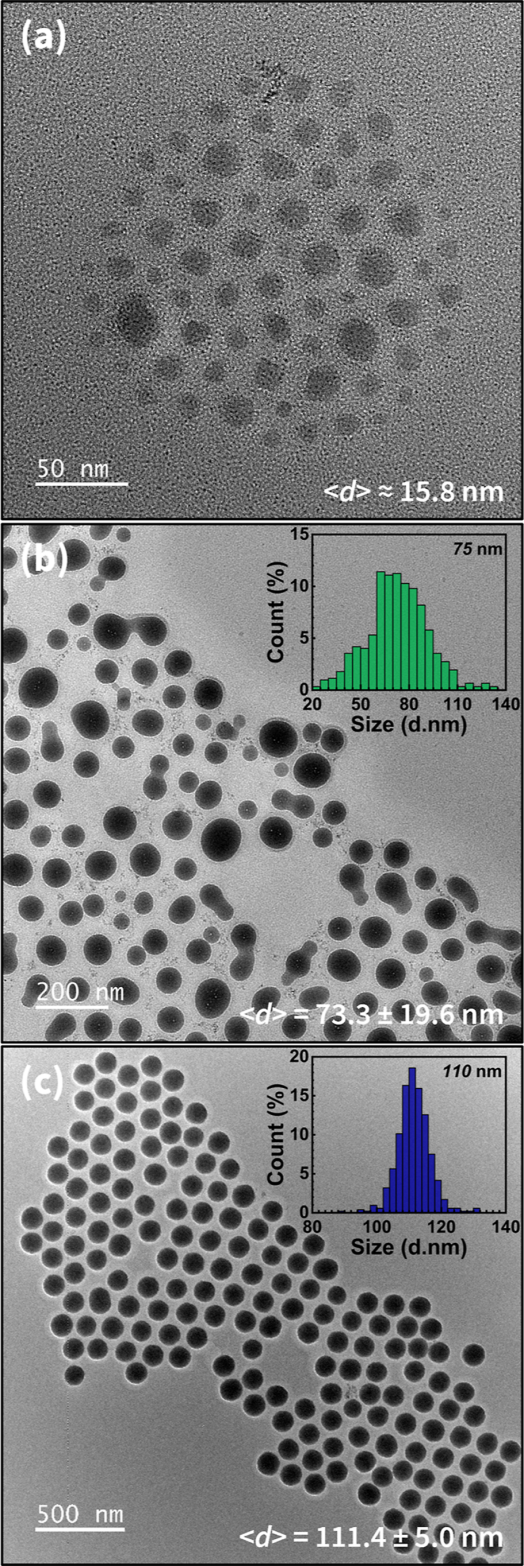
TEM images of SiO_2_-*g*-PMMA with SiO_2_ core diameters of approximately (a) 15 nm (scale bar 50 nm), (b) 75 nm (scale bar 200 nm), and (c) 110 nm (scale bar 500 nm). Inset: corresponding size distributions determined by random sampling from multiple nanoparticles.

For 75 and 110 nm SiO_2_ prepared exclusively with active initiators, σ_in_ was approximately 0.7 Br nm^–2^, consistent with that of 15 nm SiO_2_ NPs. However, when using mixed silanes with a molar ratio of active/dummy initiators of 40/30, σ_in_ was around 0.5 Br nm^–2^, which was slightly higher in comparison to that of the 15 nm SiO_2_ analog. This was attributed to the trifunctional nature of the silanes, which can provide multiple linkages, thus, providing stronger initiator anchoring and stability.

### Surface-Initiated ATRP and Grafting Density Regulation

SI-ARGET ATRP was employed to grow PMMA brushes from the surface-modified SiO_2_–Br,^49–51^ starting with the 15 nm diameter particles. Polymerizations were conducted in homogeneous dispersions using anisole as a solvent, while limiting the overall monomer conversion (<10%) to minimize interparticle brush coupling and avoid macroscopic gelation.^46,52^ Monomer conversion was estimated using a gravimetric method, coupled with the variation of inorganic fraction determined by TGA (eqs S5–S17 and Table S3). The initial monomer-to-macroinitiator ratio was fixed at [MMA]_0_/[SiO_2_–Br]_0_ = 5000:1. This means that for SiO_2_–Br with lower σ_in_, a larger amount of SiO_2_–Br was introduced to maintain a constant total initiator concentration in the reaction mixture. The absolute molecular weight (*M*_n,abs_) and molecular weight distribution (*M*_w_/*M*_n_) of grafted PMMA were determined by GPC after cleaving the polymers from the SiO_2_ cores using hydrofluoric acid, followed by neutralization with aqueous ammonia and collection of the cleaved PMMA from the THF phase.

Previous studies have shown that both the *M*_w_/*M*_n_ of grafted brushes and the σ of PGNPs can be tuned by adjusting the initial concentration of Cu catalyst ([Cu^II^Br_2_]_0_).^44^ As the Cu catalyst influences both initiation and activation/deactivation of propagating chains in ATRP, in this series of experiments, [Cu^II^Br_2_]_0_ was decreased by about 3-fold (half the order of magnitude) between successive polymerizations (Figure 1c). Lower catalyst concentrations resulted in less efficient initiation (due to slower deactivation) and nonuniform brush growth,^53–55^ which both reduced σ. The polymerizations were performed on SiO_2_–Br with four distinct initiator densities (σ_in_ = 0.7, 0.4, 0.1, and 0.03 Br nm^–2^). To mitigate oxygen inhibition, particularly critical at ultralow [Cu^II^Br_2_]_0_ (e.g., 1 and 0.3 ppm relative to monomer), a constant concentration of reducing agent – tin­(II) 2-ethylhexanoate (Sn­(Oct)_2_) was included in the reaction mixtures (used in an excess).

### PMMA Grafting from 15 nm SiO_2_–Br with Varied Initiator Density

Following SI-ARGET ATRP from 15 nm SiO_2_–Br, the resulting SiO_2_-*g*-PMMA samples were comprehensively characterized (Table 1). The measured parameters included *M*_n,abs_ and *M*_w_/*M*_n_ of the grafted PMMA by GPC analysis, inorganic weight fraction (*f*_inorg_) determined by TGA, as well as the calculated grafting density (σ) and initiation efficiency (*I*_eff_).

**Table 1:** SiO_2_-*g*-PMMA (*d*_silica_ ≈ 15 nm) Prepared via SI-ARGET ATRP with Varied Initiator Density and Cu Catalyst Concentration

entry^a^			*M*_n,abs_ ^b^ (×10^3^)	*M*_w_/*M*_n_ ^b^	*f*_inorg_ ^c^ (%)	σ^d^ (chains nm^–2^)	*I*_eff_ ^e^ (%)
*d* (nm)	σ_in_ (Br nm^–2^)	[Cu^II^Br_2_]_0_ (ppm)					
**15**	**0.7**	**100**	20.4	1.23	21	0.662	95
**15**	**0.7**	**30**	53.4	1.15	10	0.565	81
**15**	**0.7**	**10**	53.2	1.19	12	0.496	71
**15**	**0.7**	**3**	41.7	1.20	16	0.434	62
**15**	**0.7**	**1**	77.2	1.66	17	0.224	32
**15**	**0.7**	**0.3**	190.3	2.43	24	0.060	9
**15**	**0.4**	**100**	23.3	1.18	27	0.406	102
**15**	**0.4**	**30**	39.9	1.18	17	0.427	107
**15**	**0.4**	**10**	60.6	1.20	14	0.340	85
**15**	**0.4**	**3**	86.6	1.66	26	0.113	28
**15**	**0.4**	**1**	240.8	2.32	31	0.032	8
**15**	**0.4**	**0.3**	234.2	2.56	30	0.034	8
**15**	**0.1**	**100**	47.8	1.13	44	0.093	93
**15**	**0.1**	**30**	40.8	1.19	54	0.073	73
**15**	**0.1**	**10**	44.6	1.16	47	0.087	87
**15**	**0.1**	**3**	25.6	1.26	83	0.027	27
**15**	**0.1**	**1**	65.8	1.72	77	0.016	16
**15**	**0.1**	**0.3**	231.2	2.29	78	0.004	4
**15**	**0.03**	**100**	51.1	1.10	70	0.030	99
**15**	**0.03**	**30**	37.2	1.23	79	0.026	86
**15**	**0.03**	**10**	34.7	1.25	87	0.015	51
**15**	**0.03**	**3**	35.1	1.57	94	0.006	21
**15**	**0.03**	**1**	87.0	2.33	91	0.004	13
**15**	**0.03**	**0.3**	253.6	2.61	84	0.003	9

a Reaction conditions: MMA 3 mL (50 vol % in anisole), [MMA]_0_/[15 nm SiO_2_–Br]_0_/[Sn­(Oct)_2_] = 5000:1:3, [Cu^II^Br_2_]_0_/[Me_6_TREN]_0_ = 1:5, Cu^II^Br_2_ (stock solutions in *N*,*N*-dimethylformamide, DMF) 100/30/10/3/1/0.3 ppm (relative to MMA).

b Absolute molecular weight and molecular weight distribution determined by THF GPC using PMMA standards.

c Inorganic weight fraction determined by TGA under air.

d Grafting density calculated using eq 1.

e Initiation efficiency calculated as σ/σ_in_.

Besides the Cu catalyst concentration, the initiator spacing also influenced the σ of SiO_2_-*g*-PMMA (Figure 3a). For SiO_2_–Br with the highest initiator density (σ_in_ = 0.7 Br nm^–2^), σ decreased continuously as [Cu^II^Br_2_]_0_ was lowered, consistent with previous reports on grafting density regulation.^44^ When the initiator spacing was slightly increased (σ_in_ = 0.4 Br nm^–2^), these SiO_2_–Br exhibited tolerance to reduced Cu levels, maintaining σ ≈ 0.4 chains nm^–2^ at 30 ppm of Cu. This stabilizing effect became more pronounced for σ_in_ = 0.1 Br nm^–2^, where σ remained at 0.087 chains nm^–2^ even at 10 ppm of Cu. For the smallest initiator coverage (σ_in_ = 0.03 Br nm^–2^), σ still showed relative resistance to decreasing [Cu^II^Br_2_]_0_, although with a gradual decline from an already low baseline. These observations suggested that at lower σ_in_, the reduced Cu levels may still satisfy the minimum requirements for controlled polymerization and uniform brush growth. At lower σ_in_, the larger average spacing (thus a lower steric hindrance) between initiation sites could enhance the probability of successful initiation and subsequent propagation within the available reaction volume, even when catalyst levels are reduced and the overall initiation is less concurrent.

**Figure 3:**
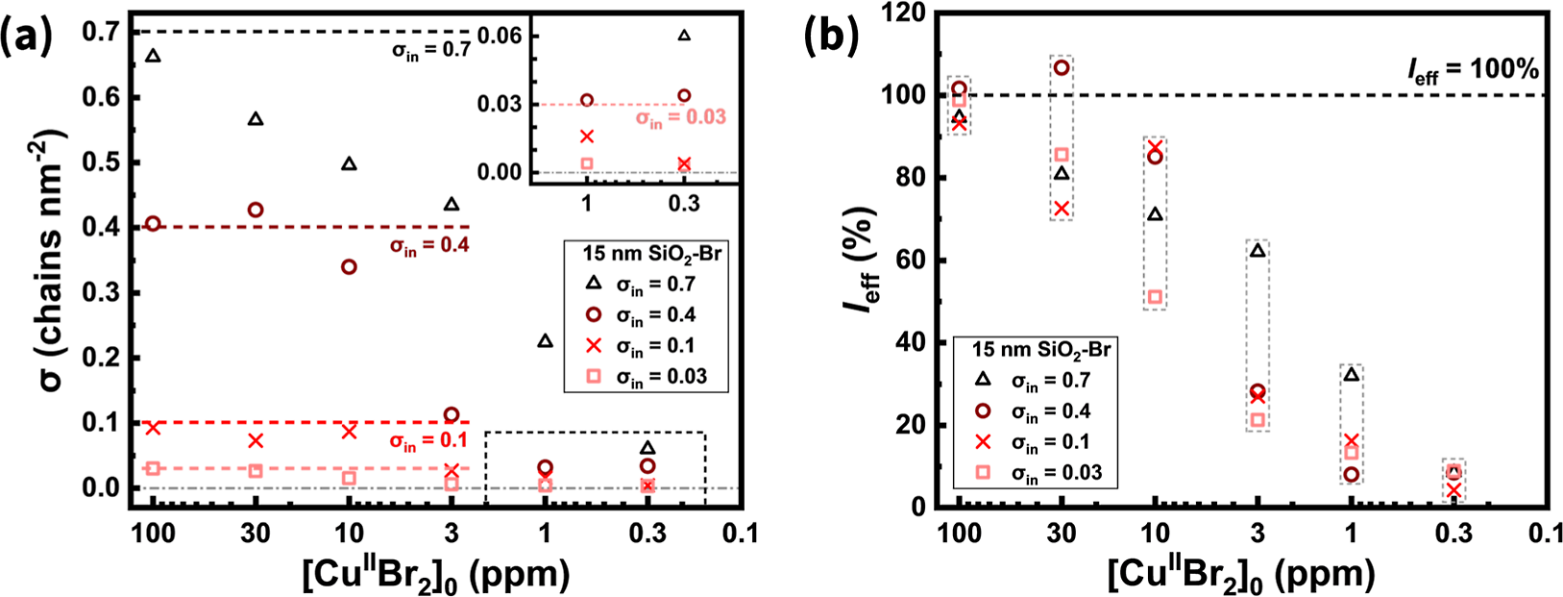
PMMA grafting from SiO_2_–Br nanoparticles (*d*_small_ ≈ 15 nm) via SI-ARGET ATRP at varied initiator density (σ_in_) and initial Cu catalyst concentrations ([Cu^II^Br_2_]_0_). Plotted parameters include: (a) grafting density (σ) and (b) initiation efficiency (*I*_eff_) as functions of [Cu^II^Br_2_]_0_. Dashed lines are included to guide the eye, representing (a) the designated σ_in_ of SiO_2_–Br and (b) the 100% initiation efficiency threshold (black) for samples prepared under identical [Cu^II^Br_2_]_0_ (gray).

Unlike polymerizations targeting unattached chains, where *I*_eff_ is typically estimated by comparing theoretical and measured molecular weights, *I*_eff_ of SiO_2_-*g*-PMMA can be approximated as the ratio of the polymer grafting density to initiator density, σ/σ_in_ (Figure 3b). This definition compares the surface density of successfully grafted polymer chains to the surface bromide density of the corresponding SiO_2_–Br macroinitiator, with the latter estimated from a model SI-ARGET ATRP experiment conducted under conditions affording near-quantitative initiation. At 100 ppm of Cu, all samples exhibited *I*_eff_ nearly 100%, indicating that SI-ARGET ATRP proceeded in a well-controlled manner with nearly complete initiator activation. As [Cu^II^Br_2_]_0_ decreased, the average *I*_eff_ followed a declining trend, reaching a value as low as ∼7% at 0.3 ppm of Cu (Figure S5). This decrease suggested that a sufficient concentration of Cu catalyst is critical for achieving the concurrent initiation of surface-anchored initiators. Insufficient catalyst contents led to nonconcurrent initiation and poor deactivation caused by the excess reducing agent, which in turn resulted in inaccessible surface bromides likely due to steric interference from surrounding, already-growing polymer chains.

The *M*_w_/*M*_n_ for both unattached chains and polymer brushes grafted from nanoparticles plays a key role in determining the physical properties and assembly behavior of polymeric materials.^56–59^ For unattached chains synthesized by (ideal) ATRP, neglecting termination, *M*_w_/*M*_n_ can be estimated from^58^

2 $$\frac{M_{w}}{M_{n}}=1+\frac{1}{{DP}_{n}}+\left(\frac{k_{p}{\left[R-X\right]}_{0}}{k_{d}\left[{Cu}^{II}Br\left(ligand\right)\right]}\right)\left(\frac{2}{\%\;conv}-1\right)$$

where DP_n_ is the degree of polymerization, [R–X]_0_ is the initial alkyl halide concentration, and *k*_p_ and *k*_d_ are the rate coefficients for propagation and deactivation, respectively.

As expected, the *M*_w_/*M*_n_ of grafted PMMA increased as [Cu^II^Br_2_]_0_ decreased (Figure 4a). At 100 ppm of Cu, where SI-ARGET ATRP was well-controlled, both high- and low-σ_in_ samples exhibited uniform brush growth (*M*_w_/*M*_n_ ≤ 1.23). Remarkably, this narrow *M*_w_/*M*_n_ was maintained even at 10 ppm of Cu, despite the concurrent decline in σ. This indicates that while initiation became less efficient, chain growth remained uniform. According to eq 2, the persistence of low *M*_w_/*M*_n_ values stems from a relatively small [R–Br]_0_/[Cu^II^Br­(Ligand)] ratio. For grafted brushes, the observed decrease in *I*_eff_ at 10 ppm of Cu implies that sterically hindered initiation or propagation sites likely became inaccessible under reduced Cu levels. At lower [Cu^II^Br_2_]_0_, the “effective” alkyl bromide concentration [R–Br] decreased, meaning that each active chain end is paired with a higher proportion of Cu. This behavior contrasts sharply with polymerizations of unattached chains, where [R–Br] remains constant by using a small-molecule initiator. Such observations highlight a fundamental mechanistic distinction between grafting-from and the corresponding processes of unattached systems and may suggest a new approach for the *M*_w_/*M*_n_ control in surface-initiated polymerizations.

**Figure 4:**
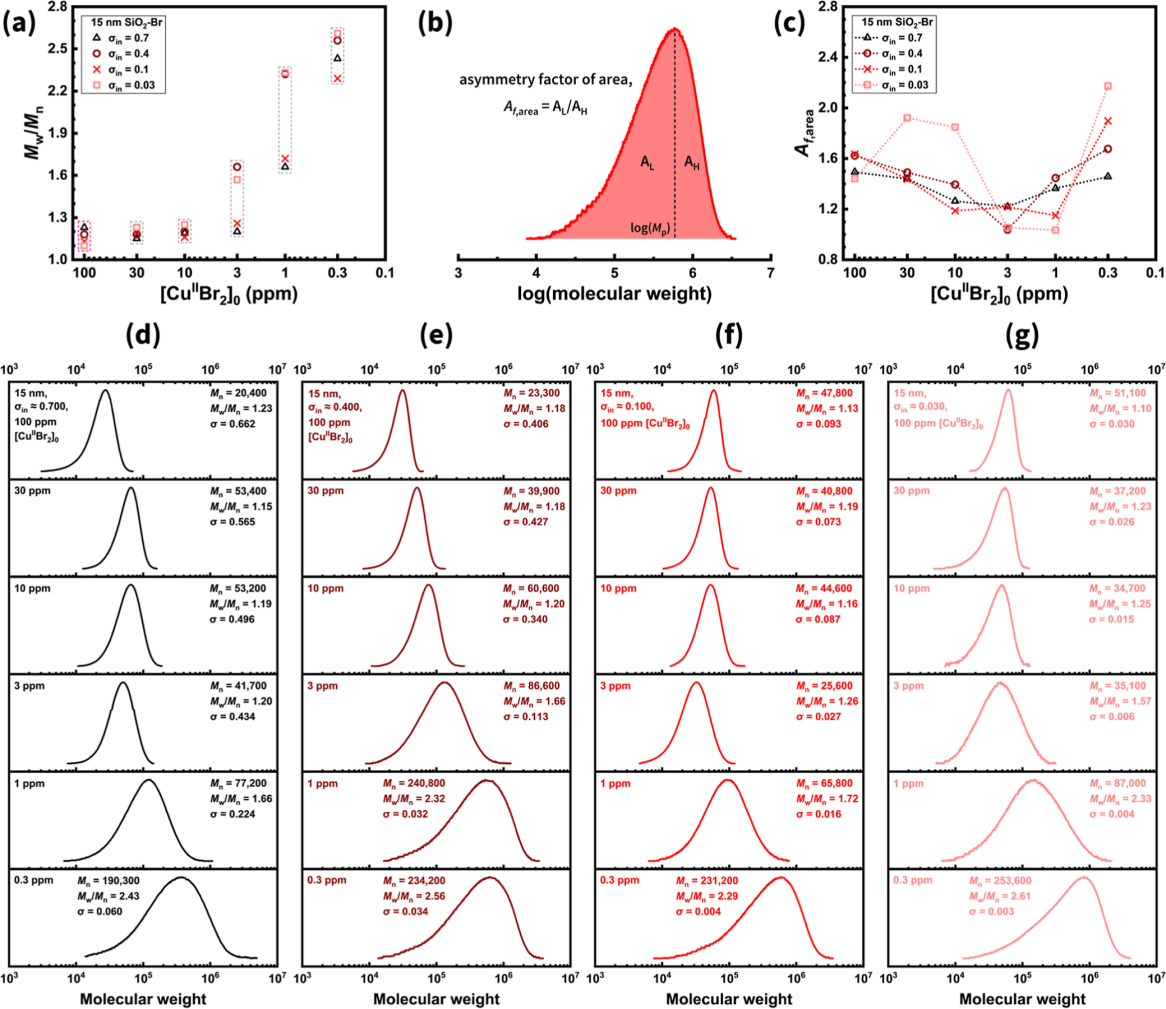
PMMA grafting from SiO_2_–Br nanoparticles (*d*_small_ ≈ 15 nm) via SI-ARGET ATRP at varied initiator density (σ_in_) and initial Cu catalyst concentrations ([Cu^II^Br_2_]_0_). (a) Molecular weight distribution (*M*_w_/*M*_n_) as a function of [Cu^II^Br_2_]_0_. Dashed lines are included to guide the eye, representing samples prepared under identical [Cu^II^Br_2_]_0_. (b) Schematic illustration of asymmetry factor calculation. (c) Asymmetry factor (*A*_f,area_) as a function of [Cu^II^Br_2_]_0_. (d–g) Normalized GPC curves of weight-average molecular weight (*M*_w_) for SiO_2_-*g*-PMMA with σ_in_ = (d) 0.7, (e) 0.4, (f) 0.1, and (g) 0.03 Br nm^–2^. In each panel, [Cu^II^Br_2_]_0_ was systematically decreased (100/30/10/3/1/0.3 ppm relative to the monomer) from top to bottom.

Given that all polymerizations were stopped at low conversions, the observed brush uniformity with sufficient Cu (at 100 ppm) was unexpected (eq 2). This was likely due to the localized nature of the surface-anchored initiators with the SiO_2_ core providing a spatially consistent template for ATRP initiation. Interestingly, at 100 ppm of Cu, brush uniformity improved as σ_in_ decreased: *M*_w_/*M*_n_ fell from 1.23 (at σ_in_ = 0.7 Br nm^–2^) to 1.10 (at σ_in_ = 0.03 Br nm^–2^). This trend suggests that at higher initiator density with sufficient Cu, crowding during propagation may introduce steric hindrance, resulting in a slightly broader *M*_w_/*M*_n_. Nevertheless, this effect was absent at lower [Cu^II^Br_2_]_0_, where reduced control resulted in higher average *M*_w_/*M*_n_ values and greater variability among samples prepared at the same Cu catalyst concentration (Figure S6).

Beyond the *M*_w_/*M*_n_ values, the shape parameters of the *M*_w_/*M*_n_, including its skewness and kurtosis, provide additional insights into the characteristics of grafted polymers. To empirically assess the distribution asymmetry, an asymmetry factor based on the integrated area (*A*_f,area_) was calculated by dividing the area to the left and right of the peak position of the weight-average molecular weight distribution (Figure 4b).^60^ Values of *A*_f,area_ > 1 indicate that the distribution is skewed toward higher molecular weight (MW), with a pronounced low-molecular-weight tailing. Representative curves of weight (*M*_w_, Figure 4d–g) and number-average molecular weight (*M*_n_, Figure S7) distributions were determined by THF GPC, respectively.

For SiO_2_-*g*-PMMA with 15 nm cores, all samples exhibited *A*_f,area_ > 1, indicating a greater fraction of polymers with MW lower than the peak position (Figure 4c). At 100 ppm of Cu, all SiO_2_-*g*-PMMA samples with different σ_in_ displayed similar asymmetry factors of ∼1.5, suggesting an intrinsically asymmetric growth profile under sufficient Cu catalyst conditions. As [Cu^II^Br_2_]_0_ decreased, a V-shaped dependence of *A*_f,area_ was observed for all σ_in_, with optimal symmetry reached at 3 ppm of Cu (Figure S8).

When the Cu level was reduced to 0.3 ppm, the skewness increased again, resulting in a pronounced low-molecular-weight tail. This phenomenon can be attributed to the following factors. (1) The excessive amount of reducing agent (600 ppm of Sn­(Oct)_2_ versus 0.3 ppm of Cu) likely led to negligible content of Cu­(II) deactivators, resulting in over-reduction and insufficient deactivation. (2) Under ultralow σ < 0.06 chains nm^–2^, where effectively initiated sites are particularly scarce, a fraction of chains remained continuously active and propagated within a larger free volume, whereas the other polymer grafts became terminated. Evidence for this lies in the more pronounced skewness observed at lower σ_in_, where only ∼2 chains per particle were grafted (σ = 0.003 chains nm^–2^ for σ_in_ = 0.03 Br nm^–2^ at 0.3 ppm of Cu).

### PMMA Grafting from 75 and 110 nm SiO_2_–Br with Varied Initiator Density

The specific surface area of a spherical nanoparticle decreases with increasing *d*, meaning that SiO_2_–Br with larger *d* provides fewer surface bromides under the same mass. Maintaining constant [SiO_2_–Br]_0_ under these conditions would require substantially higher macroinitiator loadings. To mitigate the resulting viscosity of the SI-ARGET ATRP system, PMMA grafting from larger SiO_2_–Br was therefore conducted at a lower initiator concentration ([MMA]_0_/[SiO_2_–Br]_0_ = 9000:1), while keeping all other reaction conditions constant. The reduced [SiO_2_–Br]_0_ was maintained above 100 ppm relative to the monomer concentration, thereby preventing the decrease in grafting density that was observed at lower initiator loadings.^52^ The resulting 75 and 110 nm SiO_2_-*g*-PMMA samples were comprehensively characterized, with the results summarized in Table 2.

**Table 2:** SiO_2_-*g*-PMMA (*d*_silica_ ≈ 75 and 110 nm) Prepared via SI-ARGET ATRP with Varied Initiator Density and Cu Catalyst Concentration

entry^a^			*M*_n,abs_ ^b^ (×10^3^)	*M*_w_/*M*_n_ ^b^	*f*_inorg_ ^c^ (%)	σ^d^ (chains nm^–2^)	*I*_eff_ ^e^ (%)
*d* (nm)	σ_in_ (Br nm^–2^)	[Cu^II^Br_2_]_0_ (ppm)					
**75**	**0.7**	**100**	40.0	1.22	40	0.615	88
**75**	**0.7**	**30**	62.7	1.20	30	0.615	88
**75**	**0.7**	**10**	52.3	1.29	37	0.518	74
**75**	**0.7**	**3**	65.3	1.59	40	0.370	53
**75**	**0.7**	**1**	135.0	2.78	49	0.123	18
**75**	**0.7**	**0.3**	52.9	4.12	79	0.081	12
**75**	**0.5**	**100**	37.9	1.23	48	0.470	94
**75**	**0.5**	**30**	46.0	1.28	43	0.476	95
**75**	**0.5**	**10**	36.8	1.32	56	0.351	70
**75**	**0.5**	**3**	77.7	2.04	51	0.202	40
**75**	**0.5**	**1**	127.6	2.04	52	0.116	23
**75**	**0.5**	**0.3**	73.5	4.09	68	0.104	21
**110**	**0.7**	**100**	39.4	1.26	49	0.646	92
**110**	**0.7**	**30**	29.7	1.23	62	0.509	73
**110**	**0.7**	**10**	37.2	1.34	58	0.474	68
**110**	**0.7**	**3**	41.4	1.41	65	0.325	46
**110**	**0.7**	**1**	72.8	2.30	60	0.221	32
**110**	**0.7**	**0.3**	112.1	2.87	72	0.085	12
**110**	**0.5**	**100**	51.7	1.30	49	0.491	98
**110**	**0.5**	**30**	59.9	1.28	44	0.524	105
**110**	**0.5**	**10**	54.0	1.37	51	0.446	89
**110**	**0.5**	**3**	74.8	1.70	58	0.241	48
**110**	**0.5**	**1**	132.4	2.54	59	0.128	26
**110**	**0.5**	**0.3**	55.8	3.12	77	0.131	26

a Reaction condition: MMA 3 mL (50 vol % in anisole), [MMA]_0_/[75 or 110 nm SiO_2_–Br]_0_/[Sn­(Oct)_2_] = 9000:1:5.4, [Cu^II^Br_2_]_0_/[Me_6_TREN]_0_ = 1:5, Cu^II^Br_2_ (stock solutions in DMF) 100/30/10/3/1/0.3 ppm (relative to MMA).

b Absolute molecular weight and molecular weight distribution determined by THF GPC using PMMA standards.

c Inorganic weight fraction determined by TGA under air.

d Grafting density calculated using eq 1.

e Initiation efficiency calculated as σ/σ_in_.

Compared to PMMA grafted from a 15 nm SiO_2_ analogue, a similar decrease was observed in the grafting density of SiO_2_-*g*-PMMA with varied σ_in_ as [Cu^II^Br_2_]_0_ decreased (Figure 5a). For larger SiO_2_–Br (for both *d* = 75 and 110 nm) with the highest σ_in_ of 0.7 Br nm^–2^, σ decreased continuously as the Cu catalyst concentration was lowered. When the initiator spacing slightly increased (σ_in_ = 0.5 Br nm^–2^), the nanoparticles exhibited tolerance to reduced Cu concentrations: σ remained ∼0.5 chains nm^–2^ at 30 ppm of Cu before gradually declining at lower [Cu^II^Br_2_]_0_. All samples exhibited ∼90% *I*_eff_ at 100 ppm of Cu (Figure 5b). In contrast, as [Cu^II^Br_2_]_0_ decreased, the average *I*_eff_ declined, consistent with the trend observed for the 15 nm SiO_2_–Br system. Notably, when more than 10 ppm of Cu was applied in the reaction mixture, SiO_2_–Br of the same size but with larger initiator spacing (σ_in_ = 0.5 Br nm^–2^) displayed an overall higher *I*_eff_ compared to their denser counterparts (σ_in_ = 0.7 Br nm^–2^). This suggests that increasing initiator spacing (and thereby reducing site crowding) can mitigate steric hindrance from the surrounding chains, leading to a modest improvement in *I*_eff_ even under conditions of nonconcurrent initiation.

**Figure 5:**
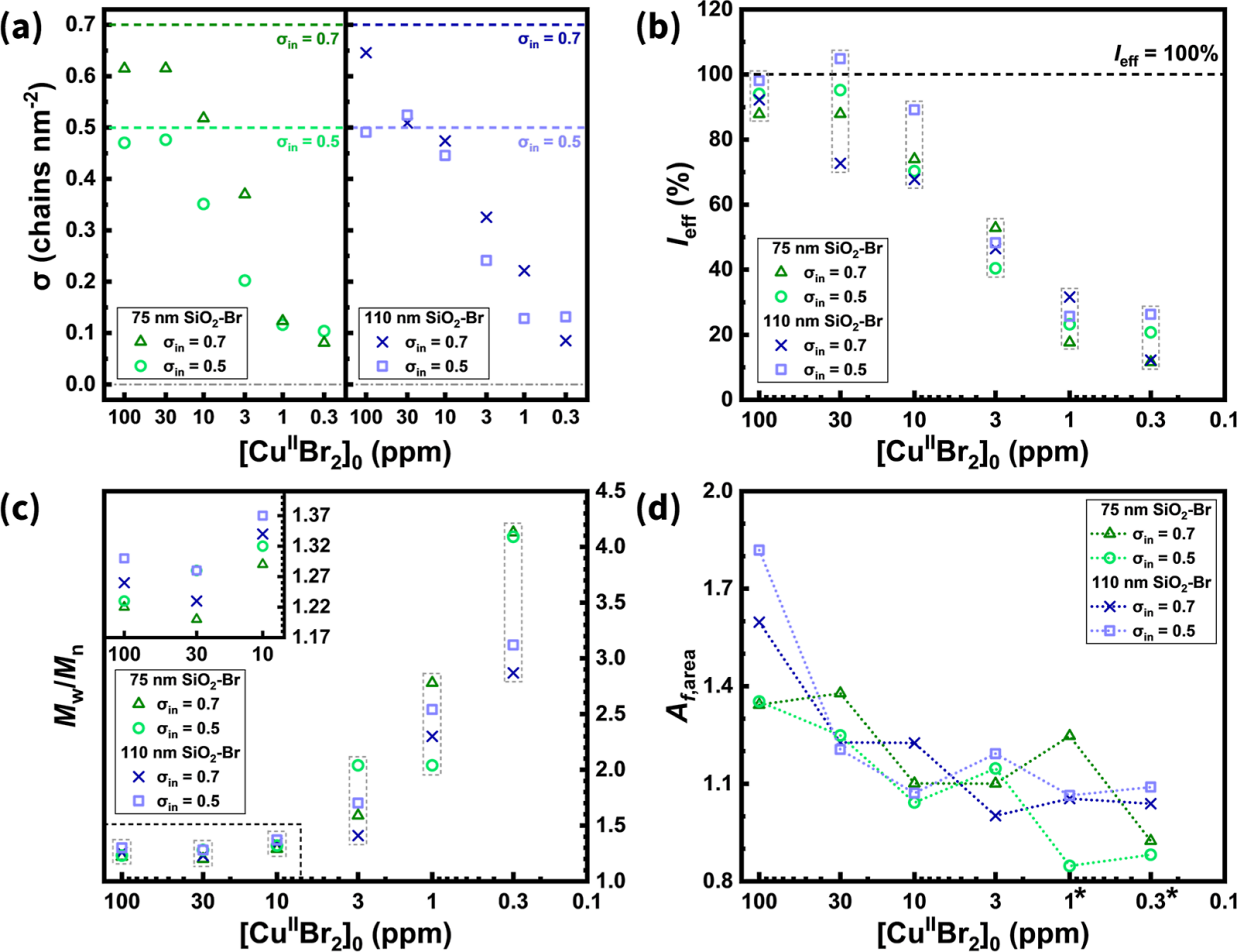
PMMA grafting from SiO_2_–Br nanoparticles (*d*_medium_ ≈ 75 nm and *d*_large_ ≈ 110 nm) via SI-ARGET ATRP at varied initiator density (σ_in_) and initial Cu catalyst concentrations ([Cu^II^Br_2_]_0_). Plotted parameters include: (a) grafting density (σ), (b) initiation efficiency (*I*_eff_), (c) molecular weight distribution (*M*_w_/*M*_n_), and (d) asymmetry factor (*A*_f,area_) as functions of [Cu^II^Br_2_]_0_. Dashed lines are included to guide the eye, representing (a) the designated σ_in_ of SiO_2_–Br, (b) the 100% initiation efficiency threshold (black), and (b,c) samples prepared under identical [Cu^II^Br_2_]_0_ (gray). The asterisks in (d) denote *A*_f,area_ values that include contributions from continuously propagated chains without deactivation.

The overall *M*_w_/*M*_n_ of grafted PMMA increased as [Cu^II^Br_2_]_0_ decreased (Figure 5c). When Cu levels above 10 ppm were applied, SiO_2_–Br with the largest *d* of 110 nm exhibited higher *M*_w_/*M*_n_ values compared to the 75 nm analog. This effect is attributed to reduced surface curvature at larger particle sizes, which increases the chain crowdedness and compromises brush length uniformity. Notably, under these conditions, SiO_2_–Br with a larger initiator spacing (σ_in_ = 0.5 Br nm^–2^) displayed broader molecular weight distributions, in contrast to the behavior observed for 15 nm SiO_2_–Br at 100 ppm of Cu. A plausible explanation considers both *I*_eff_ and the constant initial bromide concentration across the reactions. SiO_2_–Br with σ_in_ = 0.5 Br nm^–2^ illustrates a higher *I*_eff_, thereby increasing the effective concentration of alkyl bromide during the propagation stage. Consequently, the available Cu catalyst is distributed across a greater number of chain ends, reducing the catalyst-to-chain ratio and resulting in a broader *M*_w_/*M*_n_.

When [Cu^II^Br_2_]_0_ decreased below 3 ppm, *M*_w_/*M*_n_ increased significantly, with the effect most pronounced at the ultralow level of 0.3 ppm. Although it is barely apparent in the *M*_n_ data (Figure S9), GPC traces of the PMMA brush layers revealed bimodal distributions, with a secondary population of much longer chains than those in the principal peak (Figure 6). At such low [Cu^II^Br_2_]_0_, the large excess of reducing agent likely reduced nearly all Cu^II^Br_2_ deactivator species, resulting in very poor deactivation and leaving most propagating chain ends continuously active. In addition, the broad *M*_w_/*M*_n_ combined with low grafting density likely contributed to brush inhomogeneity (especially for 75 nm SiO_2_-*g*-PMMA with broad *d* distribution), further increasing *M*_w_/*M*_n_ and driving the final values above 4.

**Figure 6:**
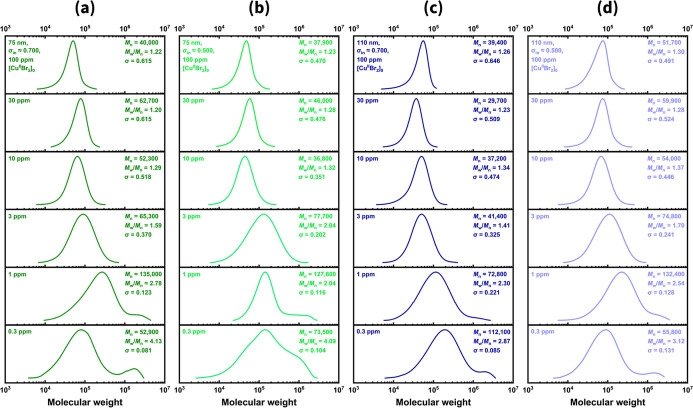
Normalized GPC analysis of the weight-average molecular weight (*M*_w_) of PMMA grafting from SiO_2_–Br, with diameter and σ_in_ of (a) 75 nm, σ_in_ = 0.7 Br nm^–2^, (b) 75 nm, σ_in_ = 0.5 Br nm^–2^, (c) 110 nm, σ_in_ = 0.7 Br nm^–2^, and (d) 110 nm, σ_in_ = 0.5 Br nm^–2^. In each panel (from top to bottom), [Cu^II^Br_2_]_0_ was systematically decreased (100/30/10/3/1/0.3 ppm relative to monomer) for polymerizations.

At sufficient Cu levels, SiO_2_-*g*-PMMA with larger core sizes exhibited higher *A*_f,area_ values, attributable to an increased chain crowding (Figure 5d). For SiO_2_–Br with a larger size (lower surface curvature), the densely packed chains were expected to generate a pronounced steric hindrance. As [Cu^II^Br_2_]_0_ decreased, *A*_f,area_ generally followed a downward trend. In this regime, sterically hindered chains propagated less efficiently, whereas unconfined chains continued to grow, leading to *A*_f,area_ approaching unity at ∼3 ppm. However, under ultralow Cu conditions (1 and 0.3 ppm), the bimodal molecular weight distributions arising from inhomogeneity were also included in the *A*_f,area_ calculation, making such values less representative.

### Comparison of SiO_2_-*g*-PMMA with Different Curvatures and Unattached PMMA

To compare the chain growth of SiO_2_-*g*-PMMA of varied surface curvature with that of unattached polymer chains, ARGET ATRP was conducted using ethyl α-bromoisobutyrate (EBiB) as the small-molecule initiator at a diluted monomer concentration (20 vol % in anisole). The [Cu^II^Br_2_]_0_ level was systematically varied, and the reactions were quenched at relatively low monomer conversions. The resulting unattached PMMA samples were characterized, including GPC analysis (Figure S10), and the results are summarized in Table 3.

**Table 3:** Unattached PMMA Prepared via ARGET ATRP under the Varied Cu Catalyst Concentration

entry^a^	[Cu^II^Br_2_]_0_ (ppm)	conv^b^ (%)	*M*_n,theo_ ^c^ (×10^3^)	*M*_n,abs_ ^d^ (×10^3^)	*M*_w_/*M*_n_ ^d^	*I*_eff_ ^e^ (%)
**L-100**	**100**	9.8	49.1	54.0	1.35	91
**L-30**	**30**	8.7	44.0	50.0	1.35	88
**L-10**	**10**	9.4	47.3	59.0	1.49	80
**L-3**	**3**	11.8	59.3	81.7	1.71	73
**L-1**	**1**	3.0	15.3	17.7	1.80	87
**L-0.3**	**0.3**	7.1	35.8	40.2	1.92	89

a Reaction condition: MMA 1.2 mL (20 vol % in anisole), [MMA]_0_/[EBiB]_0_/[Sn­(Oct)_2_] = 5000:1:3, [Cu^II^Br_2_]_0_/[Me_6_TREN]_0_ = 1:5, Cu^II^Br_2_ (stock solutions in DMF) 100/30/10/3/1/0.3 ppm (relative to MMA).

b Monomer conversion roughly estimated gravimetrically (eqs S5–S15).

c Theoretical molecular weight was calculated as *M*_n,theo_ = conv × 5000 × 100.12 + 195.05; where 195.05 corresponds to the molecular weight of EBiB.

d Absolute molecular weight and molecular weight distribution determined by THF GPC using PMMA standards.

e Initiation efficiency calculated as *M*_n,theo_/*M*_n,abs_.

Comparisons were made between unattached PMMA and all SiO_2_-*g*-PMMA samples with the highest initiator density (σ_in_ = 0.7 Br nm^–2^). A very pronounced difference is observed in their initiation efficiencies (Figure 7a). Even at low monomer conversions (<12%), unattached PMMA displayed *I*_eff_ values above 80%. Given sufficient reaction time, nearly all EBiB molecules should initiate, even as [Cu^II^Br_2_]_0_ decreased. In contrast, the *I*_eff_ of SiO_2_-*g*-PMMA consistently decreased with a reduced Cu level. Slow initiation and nonuniform chain growth left a substantial fraction of initiating sites permanently buried, screened by already growing chains, and inaccessible to be activated during polymerization.

**Figure 7:**
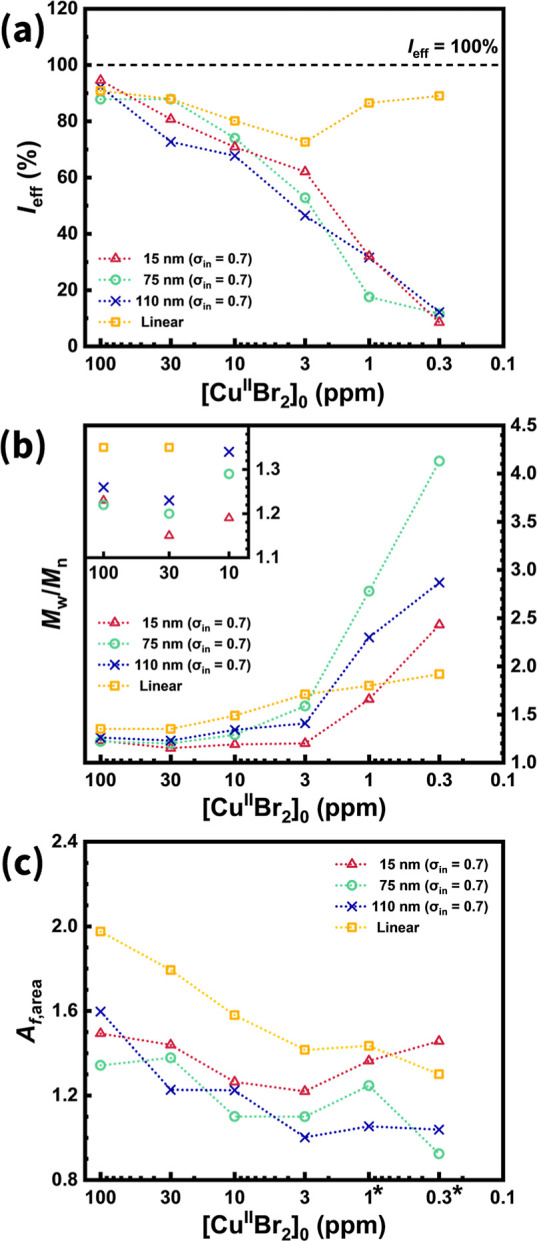
PMMA characteristics comparison of SiO_2_-*g*-PMMA with different surface curvatures and unattached PMMA via (SI-) ARGET ATRP at varied initial Cu catalyst concentrations ([Cu^II^Br_2_]_0_). Plotted parameters include: (a) initiation efficiency (*I*_eff_), (b) molecular weight distribution (*M*_w_/*M*_n_), and (c) asymmetry factor (*A*_f,area_) as functions of [Cu^II^Br_2_]_0_. Dashed lines are included to guide the eye, representing (a) the 100% initiation efficiency threshold. The asterisks in (c) denote *A*_f,area_ values (of 75 and 110 nm SiO_2_-*g*-PMMA samples) that include contributions from continuously propagated chains without deactivation.

The effect of [Cu^II^Br_2_]_0_ on *M*_w_/*M*_n_ for these systems is shown in Figure 7b. When [Cu^II^Br_2_]_0_ was above 10 ppm, unattached PMMA exhibited the highest values of *M*_w_/*M*_n_, likely due to more pronounced termination and the continuous generation of new chains, resulting from slow initiation. The grafted PMMA brushes had narrower *M*_w_/*M*_n_ because the generation of new chains was prevented by the population of already growing chains, which screened the buried initiation sites. The *M*_w_/*M*_n_ of chains grown from the smallest particles (*d* ≈ 15 nm) was the lowest. However, the *M*_w_/*M*_n_ values of polymers grown from the nanoparticles at the lowest [Cu^II^Br_2_]_0_ significantly increased. This could be due to the continuous reduction of Cu deactivators by Sn­(Oct)_2_ used at large excess (600 ppm, thereby ca. 1000 fold excess over Cu^II^Br_2_/ligand). Thus, after a certain time, there were no deactivators available to control polymerization and uncontrolled polymers with broad or even bimodal distributions and molecular weights in the range of millions were formed. Interestingly, unattached polymers had narrower *M*_w_/*M*_n_ because they were at a higher concentration (higher *I*_eff_) and could more efficiently “regenerate” Cu^II^Br_2_(ligand) deactivators by radical termination. This could also suggest that termination for SI-ARGET ATRP could be suppressed due to the segregation of radially growing chains attached to the particles. A low nanoparticle concentration, resulting in large interparticle distances, together with a higher surface curvature, which increases the radially spatial separation of growing chains, can further reduce the probability of termination. The relatively high and constant concentration of Sn­(Oct)_2_ was needed to overcome residual oxygen, but plausibly, the more careful deoxygenation and lower [Sn­(Oct)_2_] could extend control of SI-ARGET ATRP to sub-ppm catalyst level.

The asymmetry factor, *A*_f,area_, decreased with diminishing [Cu^II^Br_2_]_0_, although differences among SiO_2_-*g*-PMMA were less discernible (Figure 7c). For unattached PMMA, *A*_f,area_ remained the highest when [Cu^II^Br_2_]_0_ exceeded 1 ppm, probably due to a combination of increased termination and continuous initiation from EBiB. The former phenomenon may arise from the higher probability of encountering two propagating macroradicals, in contrast to the spatially localized, surface-bound, and radially propagating radicals for SiO_2_-*g*-PMMA. The latter effect reflects the continuous generation of new chains during polymerization, which contributed to larger *A*_f,area_ values. It should be noted that distinguishing whether the low MW fraction (*A*_L_ as in Figure 4b) originates from early termination or continuous initiation remains challenging. It will require more elaborate experimental or theoretical approaches in future studies. Collectively, these observations highlight the unique features of surface-initiated systems and underscore the role of steric hindrance in governing chain growth from SiO_2_–Br macroinitiators.

## Conclusions

This study systematically elucidated how initiator spacing, Cu catalyst concentration, and nanoparticle curvature govern the chain growth and uniformity of PMMA brushes grafted from spherical SiO_2_ nanoparticles via SI-ARGET ATRP. By tuning the initiator density (via mixed active and dummy ATRP initiators), varying the Cu catalyst concentration, and applying SiO_2_–Br macroinitiators with three distinct diameters, we demonstrated that increased initiator density and reduced surface curvature amplify steric hindrance among the grafted chains, leading to decreased initiation efficiency and broader molecular weight distributions.

Notably, comparisons with unattached PMMA using small-molecule initiators confirmed the presence of permanently buried inaccessible initiation sites, which are unique to surface-initiated systems (as previously postulated by means of simulations).^45^ Detailed investigations revealed that, under a sufficient Cu catalyst level (100 ppm relative to monomer), brush growth remained uniform across both high and low initiator densities, whereas lower catalyst concentrations accentuated nonconcurrent initiation and propagation. The “hindered dormant” effect of grafted brushes was primarily observed when the Cu level exceeded 10 ppm; below 3 ppm, however, this effect was likely masked by the excess reducing agent, which caused over-reduction and poor deactivation. While the excess reducing agent was introduced to mitigate residual oxygen, its optimization could enable future efforts toward ATRP under sub-ppm catalyst concentrations. Furthermore, the distinction between surface-grafted systems and the polymerization of unattached PMMA chains underscored the need for refined theoretical descriptions of *M*_w_/*M*_n_ in polymer brushes growing from surfaces, particularly those accounting for sterically hindered or dormant sites.

Overall, these findings enhance the mechanistic understanding of polymer-grafted nanoparticles and provide design principles for tailoring brush uniformity through the control of the initiator density, catalyst concentration, and nanoparticle curvature. Such insights are expected to guide the structural and architectural engineering of densely grafted (nanoparticle) surfaces for applications in nanocomposites, photonics, and functional coatings.

## Supplementary Material



## Notes

Experimental procedures; grafting density derivation; ^1^H NMR spectroscopy; TEM images, characterization results, and GPC analysis of PMMA-grafted SiO_2_ nanoparticles (PDF)
