# Supplementary material for: Effect of Initiator Density, Catalyst Concentration, and Surface Curvature on the Uniformity of Polymers Grafted from Spherical Nanoparticles
Source: Macromolecules. 2026 Jan 6;59(2):689–701. doi: 10.1021/acs.macromol.5c02737 (PMC12854752; doi:10.1021/acs.macromol.5c02737)
Supplement: Supplementary file 1 [file ma5c02737_si_001.pdf]

Supporting Information for

Effect of Initiator Density, Catalyst Concentration, and Surface Curvature on the Uniformity of Polymers Grafted from Spherical Nanoparticles

Rongguan Yin,[1] Hanshu Wu,[1] Xiaolei Hu,[1] Khidong Kim,[1] Francesca Lorandi,[2] Dagmar R. D'hooge,[3,4] Edmondo M. Benetti,[2] Michael R. Bockstaller,[5] and Krzysztof Matyjaszewski[1,]*

[1] Department of Chemistry, Carnegie Mellon University, Pittsburgh, Pennsylvania 15213, United States.

[2] Laboratory for Macromolecular and Organic Chemistry, Department of Chemical Sciences, University of Padova, Padova 35131, Italy

[3] Laboratory for Chemical Technology, Department of Materials, Textiles and Chemical Engineering, Ghent University, Technologiepark 125, B-9052 Zwijnaarde (Ghent), Belgium

[4] Centre for Textile Science and Engineering, Department of Materials, Textiles and Chemical Engineering, Ghent University, Technologiepark 70A, B-9052 Zwijnaarde (Ghent), Belgium

[5] Department of Materials Science and Engineering, Carnegie Mellon University, Pittsburgh, Pennsylvania 15213, United States.

Contents

S1. Experimental Section.......... 2

S2. Silica (SiO_2) Surface Modifications and Initiator Density Regulation.......... 9

S3. PMMA Grafting from 15 nm SiO_2-Br with Varied Initiator Density. 13

S4. PMMA Grafting from 75 and 110 nm SiO_2-Br with Varied Initiator Density.......... 21

S5. GPC Analysis of Unattached PMMA.......... 22

* Corresponding author. Email: km3b@andrew.cmu.edu

S1. Experimental Section.

Materials. ORGANOSILICASOL™ – colloidal silica nanoparticles (SiO_2 NPs) in organic solvent (30 wt%), MIBK-ST (d_{small} ~ 15 nm), MEK-ST-L (d_{medium} ~ 75 nm after centrifugation and surface modification), and IPA-ST-ZL (d_{large} ~ 110 nm) were kindly donated by Nissan Chemical America Corporation. Allyl alcohol (99%, Sigma-Aldrich), dichloromethane (DCM, 99%, Sigma-Aldrich), sodium bicarbonate ($NaHCO_3$, ACS grade, Fisher Chemical), α-bromoisobutyryl bromide (BiBB, 98%, Sigma-Aldrich), sodium chloride (NaCl, ACS grade, Fisher Chemical), sodium sulfate (Na_2SO_4, 99%, anhydrous, granular, Sigma-Aldrich), chlorodimethylsilane (98%, Sigma-Aldrich), Karstedt's catalyst (platinum(0)-1,3-divinyl-1,1,3,3-tetramethyldisiloxane complex solution, in xylene, Pt ~2%, Sigma-Aldrich), hexamethyldisilazane (HMDS, 99%, Sigma-Aldrich), acetone (99%, Fisher Chemical), ethanol (EtOH, anhydrous, 200 proof, Decon Laboratories), 3-(triethoxysilyl)propyl α-bromoisobutyrate (95%, Ambeed), n-hexyltriethoxysilane (98%, Ambeed), alumina (basic, Super I, 50-200 μm, Sorbtech), tin(II) 2-ethylhexanoate ($Sn(Oct)_2$, 95%, Sigma-Aldrich), anisole (99%, Sigma-Aldrich), copper(II) bromide ($Cu^{II}Br_2$, 99%, Sigma-Aldrich), tris(2-dimethylaminoethyl)amine (Me_6TREN, 99%, KOEI), *N*,*N*-dimethylformamide (DMF, 99%, Fisher Chemical), alumina (neutral, Super I, 50-200 μm, Sorbtech), methanol (MeOH, 99%, Fisher Chemical), tetrahydrofuran (THF, 99%, Fisher Chemical), toluene (99%, Fisher Chemical), 48 wt% aqueous hydrofluoric acid (HF, 99%, Sigma-Aldrich), ammonium hydroxide aqueous solution ($NH_3{\cdot}H_2O$, 28.0-30.0%, Fisher Chemical), chloroform-d ($CDCl_3$, 99%, Cambridge Isotope Laboratories), aluminum weighing dish (42 mL, Fisher Brand), and ethyl α-bromoisobutyrate (EBiB, 98%, Sigma-Aldrich), were used as received unless otherwise stated. Methyl methacrylate (MMA, Acros Organics, 99%) was passed through a column of basic alumina prior to use to remove polymerization inhibitors.

Synthesis of 3-(chlorodimethylsilyl)propyl α-bromoisobutyrate (BiBSiCl). The synthesis was carried out in two steps as outlined.

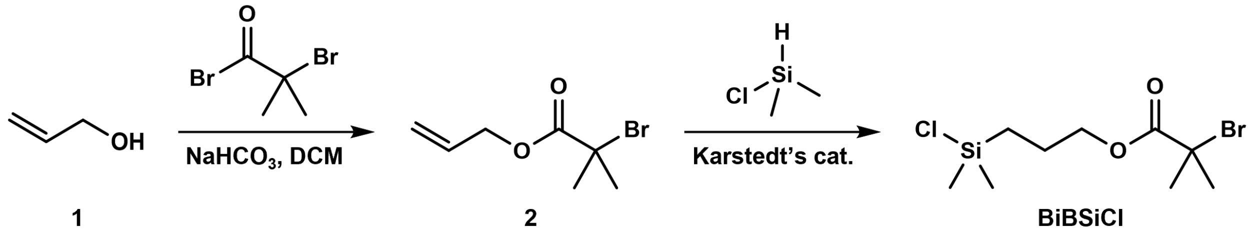

Scheme S1. Synthetic route for the "active" initiator, 3-(chlorodimethylsilyl)propyl α-bromoisobutyrate (BiBSiCl), for surface modification of 15 nm SiO_2 NP.

Step 1 – synthesis of allyl α-bromoisobutyrate (**2**). Allyl alcohol (**1**, 17 mL, 1.0 equiv) and dichloromethane (DCM, 250 mL) were charged into a 500 mL round-bottom flask. Sodium bicarbonate, ($NaHCO_3$, 42.0 g, 2.0 equiv) was added, and the mixture was cooled in an ice bath. α-Bromoisobutyryl bromide (BiBB, 33.8 mL, 1.1 equiv) was added dropwise via syringe pump at a rate of 0.5 $mL{\cdot}min^{-1}$. The reaction vessel was open to air to allow the escape of CO_2 gas. The ice bath was maintained for 2 h, after which the mixture was stirred overnight, allowing the temperature to gradually rise to room temperature. The crude product was purified by sequential liquid-liquid extractions: (i) washed three times with saturated aqueous $NaHCO_3$, (ii) washed three times with deionized water, and (iii) washed once with saturated aqueous NaCl. After each wash, the organic and aqueous layers were separated in a separatory funnel. The final organic layer was dried over anhydrous Na_2SO_4, filtered through a column of basic alumina, and the DCM was removed by a constant stream of air overnight. The purified allyl α-bromoisobutyrate (**2**) was stored at -20 °C.

Step 2 – hydrosilylation to yield BiBSiCl. Allyl α-bromoisobutyrate (**2**, 20 g, 1.0 equiv) and chlorodimethylsilane (12.3 mL, 10.5 g, 1.15 equiv) were added to a 50 mL Schlenk flask. The

reaction mixture was purged with nitrogen for 20 min, cooled in an ice bath, and Karstedt's catalyst (platinum(0)-1,3-divinyl-1,1,3,3-tetramethyldisiloxane complex solution in xylene, 0.5 mL) was added dropwise via syringe pump at 0.5 $mL \cdot h^{-1}$. The mixture was stirred for 1 h at 0 °C, then allowed to warm to room temperature over 1 h. The reaction was then heated to 60 °C in an oil bath and stirred for 24 h under nitrogen. The crude product was purified by distillation under reduced pressure to afford BiBSiCl, which was stored under vacuum in a dry environment.

Nuclear magnetic resonance (NMR) spectroscopy. The molecular scaffolds of active initiators, BiBSiCl and 3-(triethoxysilyl)propyl α-bromoisobutyrate, were analyzed by proton NMR (^1H NMR) using Bruker Avance™ III 500 MHz (^1H Frequency) NMR Instrument.

Surface modification of 15 nm SiO_2 nanoparticles. The colloidal dispersion of SiO_2 NPs in methyl isobutyl ketone (MIBK-ST, 20 mL, SiO_2 NP 30 wt% in MIBK, $d_{small} \sim 15$ nm) was added to a 50 mL round-bottom flask and purged with nitrogen for 20 min. BiBSiCl (6.0 mL, 1.0 equiv) was injected, and the reaction mixture was heated in an oil bath at 60 °C for 12 h. For tuning the initiator density (σ_{in}), active and dummy initiators were premixed at predetermined molar ratios prior to addition. After that, the reaction mixture was cooled down to room temperature. After cooling, hexamethyldisilazane (HMDS, 5.6 mL, 1.0 equiv) was added, and the mixture was refluxed at 35 °C for 12 h to cap unreacted surface silanol groups and thereby prevent SiO_2 nanoparticle aggregation. The resulting dispersion was purified by at least five cycles of dialysis (with a 10 kDa cutoff) against an acetone/methanol mixture to remove the untethered initiators. The product was dried under ambient conditions to yield SiO_2-Br, which was used directly in SI-ARGET ATRP reactions.

Surface modification of 75 and 110 nm SiO_2 nanoparticles. The colloidal dispersion of SiO_2 NPs (10 mL, 30 wt%) in methyl ethyl ketone for $d_{medium} \sim 75$ nm (MEK-ST-L, original $d \sim 50$ nm

before repetitive centrifugation), or in isopropanol for d_{large} ~ 110 nm (IPA-ST-ZL), was diluted with 40 mL ethanol (EtOH). The dispersion was subjected to high-speed centrifugation (12,000 rpm, 30 min). the supernatant was discarded, and the precipitates were immediately redispersed in EtOH with stirring. This centrifugation–redispersion process was repeated three times to achieve complete solvent exchange.

The resulting SiO_2 in EtOH dispersion (~ 50 mL) was transferred into a 500 mL round-bottom flask. Ammonium hydroxide aqueous solution ($NH_3 \cdot H_2O$, 28.0-30.0% in water, 32 mL) was diluted with EtOH (288 mL), transferred to a pressure-equalizing addition funnel, and added dropwise to the flask. After complete addition, the reaction mixture was stirred in an oil bath at 40 °C for 2 h.

The active initiator, 3-(triethoxysilyl)propyl α-bromoisobutyrate (8 g), was diluted in 24 mL EtOH, loaded into a pressure-equalizing addition funnel, and added dropwise to the flask. For tuning the initiator density (σ_{in}), active and dummy initiators were premixed at predetermined molar ratios prior to EtOH dilution. The reaction mixture was maintained at 40 °C under stirring for 12 h. The reaction mixture was concentrated under moderate air flow to remove residual aqueous ammonia, which could otherwise generate high vapor pressure and cause leakage during subsequent centrifugation. The product was purified by five cycles of centrifugation (12,000 rpm, 30 min) and redispersion in EtOH to remove unbound initiators, and then dried under ambient conditions to yield SiO_2-Br, which was used directly in SI-ARGET ATRP reactions.

High-speed centrifugation. The high-speed centrifugation was performed using a Thermo Scientific™ Sorvall™ ST16 Centrifuge equipped with a FIBERLITE™ F15-6×100y fixed-angle rotor, capable of up to 15,000 rpm or 24,700 × g.

Gel permeation chromatography (GPC). Number-average molecular weight (M_n) and molecular weight distribution (M_w/M_n) of the grafted PMMA brush layers were determined by GPC. All SiO_2-*g*-PMMA samples were etched with hydrofluoric acid (HF) for at least 12 h, neutralized with aqueous ammonia, passed through a 1 mL neutral alumina column, and filtered using a 0.45 μm PTFE filter prior to analysis. GPC measurements were performed in THF at 35 °C using a Polymer Standards Service (PSS) GPC system equipped with an Agilent 1260 Infinity II isocratic pump, a column set containing three PSS SDV analytical columns (pore sizes: 10^3 Å, 10^5 Å, 10^6 Å), and a Waters 2410 refractive index (RI) detector. The eluent (THF) was delivered at a flow rate of 1.00 $mL\cdot min^{-1}$. Data acquisition and molecular weight analysis were carried out using PSS WinGPC software (build 9666), with calibration based on PMMA standards.

Thermogravimetric analysis (TGA). TGA was performed on a TA Instrument TGA 550 under an air atmosphere, and data were processed using TA Universal Analysis software. The heating program consisted of four steps: (i) ramp from room temperature to 120 °C at 20 $°C\cdot min^{-1}$; (ii) hold at 120 °C for 10 min; (iii) high-resolution ramp to 800 °C at 20 $°C\cdot min^{-1}$; (iv) hold at 800 °C for 5 min. The organic contents of each sample were normalized to the weight loss between 120 °C and 800 °C, from which the inorganic fraction (f_{inorg}) was determined.

Transmission electron microscopy (TEM). TEM was performed on a FEI Tecnai F20 electron microscope at 200 kV. Monolayered brush particles in THF were prepared by depositing a drop of diluted solution onto carbon film-covered copper grids, followed by annealing at 100 °C for 24 h to fully equilibrate. The diameters of the SiO_2 NPs were determined by statistical analysis of TEM micrographs using MATLAB software, as described below.

SiO_2 core diameter determination. To minimize bias from manual measurements, particle sizes were determined automatically from TEM images using an in-house MATLAB script. First, the

scale factor (nm·pixel^{-1}) was calculated by inputting the real-space scale bar length (nm) and measuring its pixel length. The images were then binarized using an adaptive threshold to distinguish the SiO_2 core from the surrounding polymer brush shell. Edge detection was applied to identify all particles, and the major and minor axes of each detected particle were measured. The mean average of these two axes was taken as the particle diameter. Finally, all measurements were converted from pixels to nanometers using the scale factor, and the resulting size distributions were reported.

Initiator density (σ_{in}) determination by model reactions. Small-scale SI-ARGET ATRP model reactions were performed in triplicate to estimate the accessible σ_{in}. Reaction conditions: SiO_2-Br (0.10 g), MMA (3 mL, 50 vol% in anisole), $Cu^{II}Br_2$ (stock solution in DMF, 0.005 g·mL^{-1}) at 200 ppm relative to MMA, with $[Cu^{II}Br_2]_0$: $[Me_6TREN]_0$: $[Sn(Oct)_2]$ = 1: 3: 5, conducted at 50 °C for 30 min. Grafted PMMA was cleaved from the SiO_2 cores using HF, neutralized with aqueous ammonia, passed through a 1 mL neutral alumina column, and filtered using a 0.45 μm PTFE filter prior to analysis. The molecular weight (and distribution) was determined by THF GPC calibrated with PMMA standards. The inorganic fractions of the model particle brushes were measured by TGA. Using the molecular weight and inorganic fraction values, grafting densities were calculated according to eq S4. The average value was rounded to obtain the accessible σ_{in}, noting that actual grafting densities may vary depending on the monomer type.

Synthesis of PMMA-grafted SiO_2 nanoparticles (SiO_2-*g*-PMMA) using SI-ARGET ATRP. As an example, PMMA-grafted 110 nm SiO_2 nanoparticles with σ_{in} = 0.7 Br nm^{-2} were prepared using 100 ppm Cu. MMA (2.82 g, 3.00 mL, 50 vol% in anisole), 110 nm SiO_2 nanoparticles (109.6 mg), $Cu^{II}Br_2$ (stock solution in DMF, 0.005 g·mL^{-1}, 0.126 mL, 100 ppm relative to MMA), and Me_6TREN (stock solution in anisole, 1% v/v, 0.376 mL) were combined, followed by the addition

of anisole (~2.44 mL) to maintain a final MMA concentration of ~50 vol%. The mixture was transferred to a 10 mL Schlenk flask, sealed with a septum stopper, and purged with nitrogen for 15 min. Sn(Oct)$_2$ (fresh stock solution in anisole, 10% v/v, 0.055 mL) was then injected via syringe pump to start SI-ARGET ATRP, and the flask was immersed in an oil bath at 50 °C. Monomer conversion was determined as described (eqs S5-S17). The resulting SiO_2-*g*-PMMA was precipitated into methanol, collected after centrifugation, and redissolved in THF. Grafted PMMA was cleaved from the SiO_2 cores using HF, neutralized with aqueous ammonia, passed through a 1 mL neutral alumina column, and filtered using a 0.45 μm PTFE filter prior to analysis. The molecular weight (and distribution) was determined by THF GPC calibrated with PMMA standards. The inorganic fraction of the was determined by TGA. Using the molecular weight and inorganic fraction value, the grafting density was calculated according to eq S4.

Synthesis of unattached PMMA using ARGET ATRP. As an example, unattached PMMA was prepared using 100 ppm Cu. MMA (1.13 g, 1.20 mL, 20 vol% in anisole), EBiB (stock solution in anisole, 1% v/v, 0.033 mL), $Cu^{II}Br_2$ (stock solution in DMF, 0.005 g·mL^{-1}, 0.050 mL, 100 ppm relative to MMA), and Me$_6$TREN (stock solution in anisole, 1% v/v, 0.151 mL) were combined, followed by the addition of anisole (~4.52 mL) to maintain a final MMA concentration of ~20 vol%. The mixture was transferred to a 10 mL Schlenk flask, sealed with a septum stopper, and purged with nitrogen for 15 min. Sn(Oct)$_2$ (fresh stock solution in anisole, 5% v/v, 0.044 mL) was then injected via syringe pump to start ARGET ATRP, and the flask was immersed in an oil bath at 50 °C. Monomer conversion was determined as described (eqs S5-S15). The resulting reaction mixture (containing unattached PMMA) was diluted in THF, passed through a 1 mL neutral alumina column, and filtered using a 0.45 μm PTFE filter prior to analysis by THF GPC calibrated with PMMA standards to determine molecular weight (and distribution).

S2. Silica (SiO_2) Surface Modifications and Initiator Density Regulation.

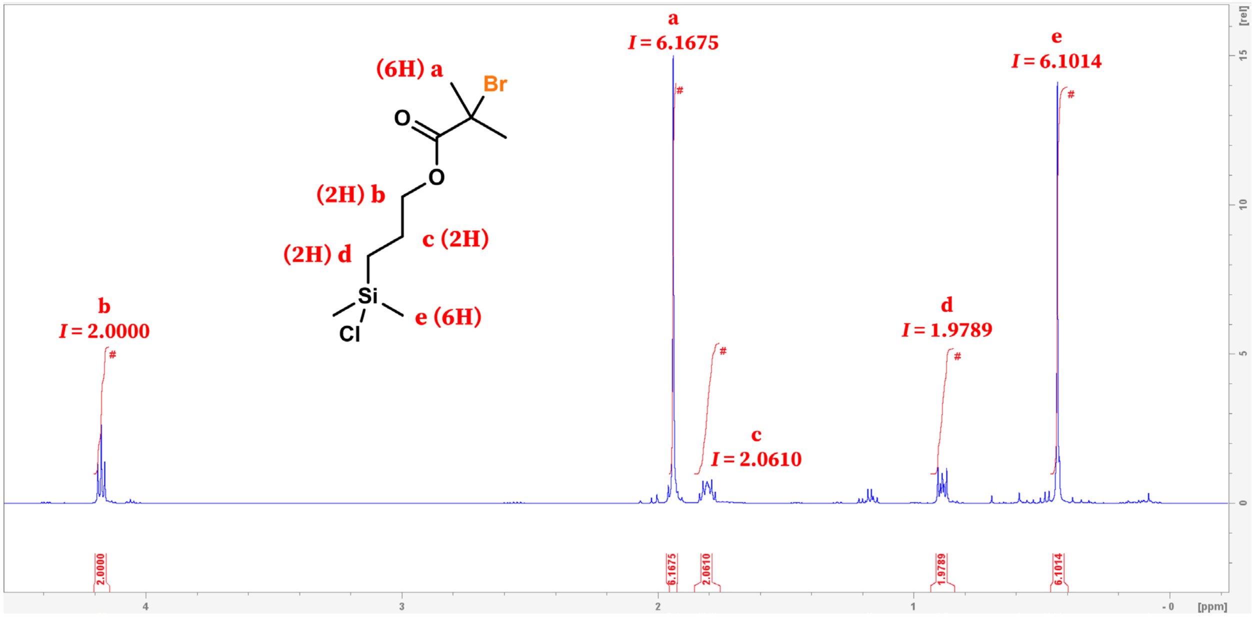

Figure S1. ^{1}H NMR spectroscopy of 3-(chlorodimethylsilyl)propyl α-bromoisobutyrate (in $CDCl_3$).

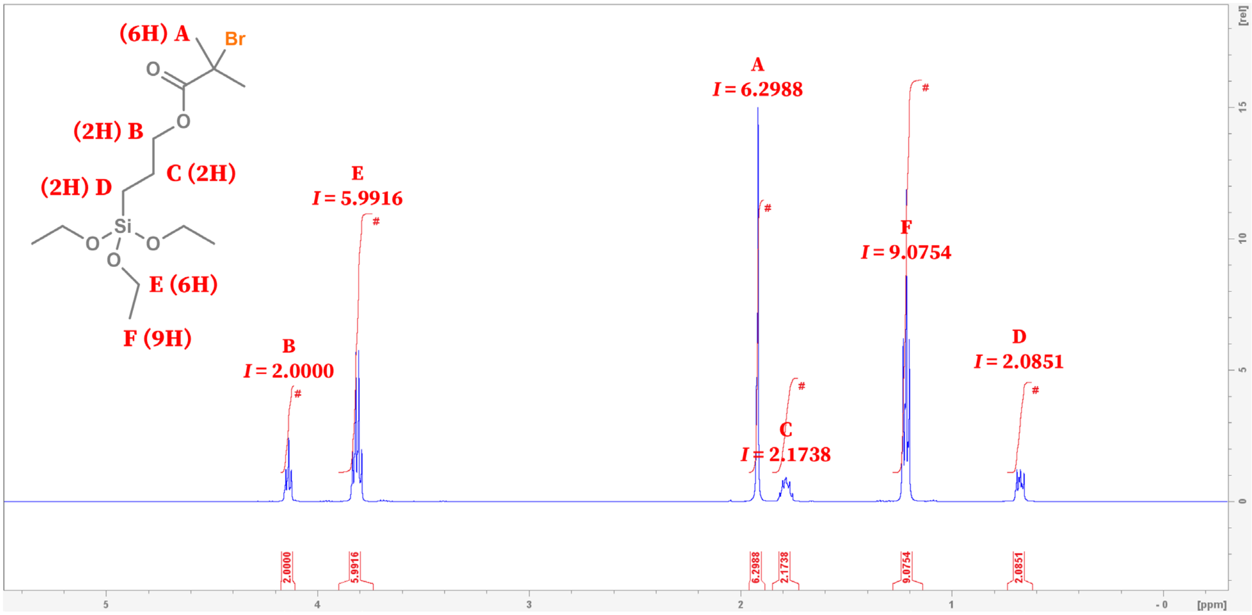

Figure S2. ^{1}H NMR spectroscopy of 3-(triethoxysilyl)propyl α-bromoisobutyrate (in $CDCl_3$).

Derivation of grafting density of SiO_2-*g*-PMMA

The grafting density (σ) is defined as the number of polymer chains per unit surface area of the spherical nanoparticle core:

$$\sigma = \frac{\text{total number of grafted PMMA chains}}{\text{total surface area of SiO}_2} \quad \text{(S1)}$$

The total number of grafted PMMA chains, N_{PMMA}, can be expressed as:

$$N_{\text{PMMA}} = \frac{m_{\text{PMMA}}\, N_{\text{A}}}{M_{\text{n}}} \quad \text{(S2)}$$

Where m_{PMMA} is the total mass (g) of grafted PMMA, N_{A} is Avogadro's constant (6.022×10^{23} mol^{-1}), and M_{n} is the number-average molar mass (g·mol^{-1}) of the grafted PMMA determined by GPC.

The specific surface area (SSA) of the spherical SiO_2 NP is given by:

$$\text{SSA} = \frac{\text{total surface area of SiO}_2}{\text{total mass of SiO}_2, m_{\text{SiO}_2}} = \frac{S}{\rho\, V} = \frac{4\pi r^2}{\rho \times \frac{4}{3}\pi r^3} = \frac{6}{\rho d} \quad \text{(S3)}$$

Where ρ is the density of SiO_2 (2.2×10^{-21} g·nm^{-3}) and d is the SiO_2 diameter. The measured core diameters were $d_{\text{small}} = 15.8$ nm, $d_{\text{medium}} = 73.3$ nm, and $d_{\text{large}} = 111.4$ nm.

By combining eqs S1 to S3 and using the inorganic weight fraction (f_{inorg}) of SiO_2-*g*-PMMA determined by TGA in air, the equation becomes:

$$\sigma = \frac{\left(\frac{m_{\text{PMMA}}\, N_{\text{A}}}{M_{\text{n}}}\right)}{m_{\text{SiO}_2}\, \text{SSA}} = \frac{m_{\text{PMMA}}}{m_{\text{SiO}_2}} \frac{N_{\text{A}}\, \rho\, d}{6\, M_{\text{n}}} = \frac{\left(1 - f_{\text{inorg}}\right) N_{\text{A}}\, \rho\, d}{6\, f_{\text{inorg}}\, M_{\text{n}}} \quad \text{(S4)}$$

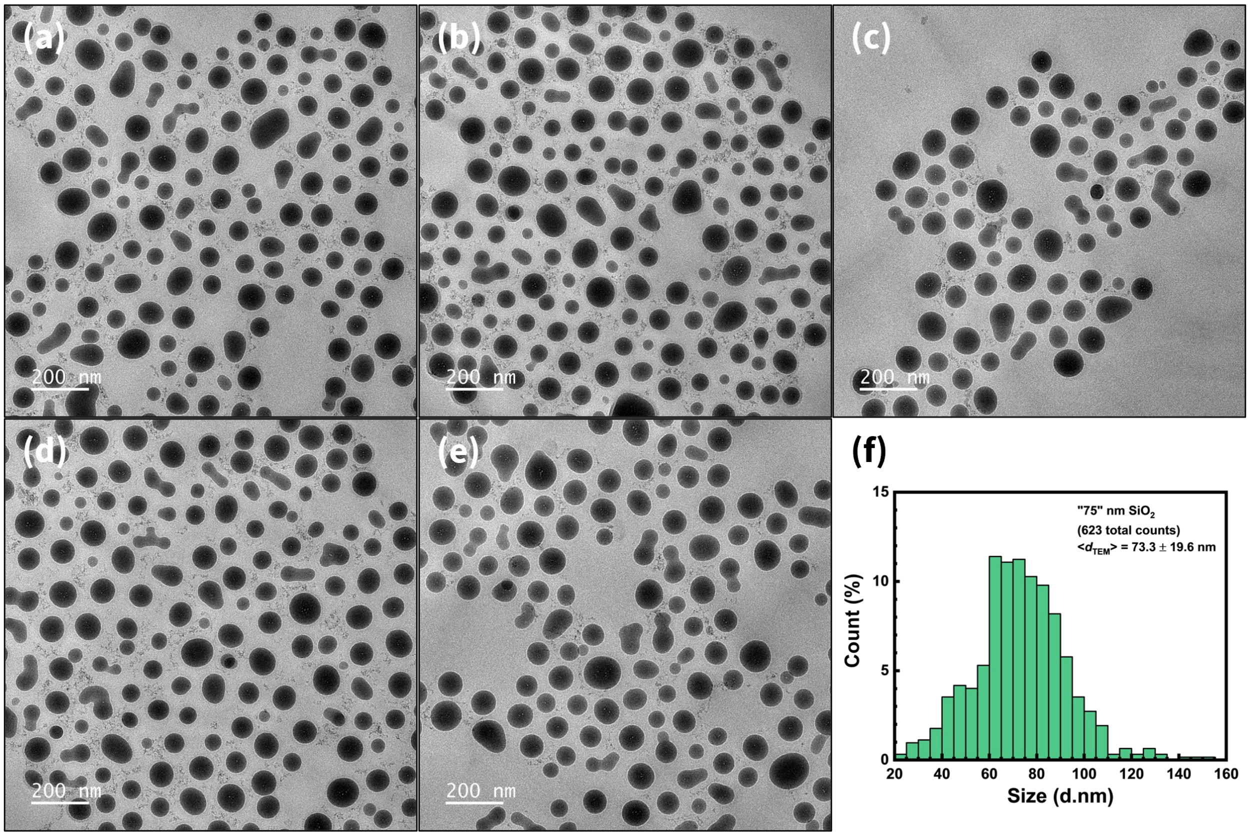

Figure S3. (a-e) Representative TEM images of SiO_2-*g*-PMMA (after model reactions) with average NP diameters of around 75 nm using a random sampling method. (f) Diameter distributions with calculated average of $d_{\mathrm{medium}} = 73.3 \pm 19.6$ nm.

Table S1. Core Diameter Distributions of SiO_2 NPs (623 total counts, *d* ~ 75 nm).

Size (nm)	Counts	Count (%)	Size (nm)	Counts	Count (%)	Size (nm)	Counts	Count (%)
20-25	2	0.3	70-75	70	11.2	120-125	2	0.3
25-30	6	1.0	75-80	64	10.3	125-130	4	0.6
30-35	7	1.1	80-85	61	9.8	130-135	2	0.3
35-40	11	1.8	85-90	51	8.2	135-140	0	0.0
40-45	22	3.5	90-95	36	5.8	140-145	1	0.2
45-50	26	4.2	95-100	22	3.5	145-150	1	0.2
50-55	25	4.0	100-105	17	2.7	150-155	1	0.2
55-60	33	5.3	105-110	12	1.9	155-160	0	0.0
60-65	71	11.4	110-115	2	0.3	160-165	1	0.2
65-70	69	11.1	115-120	4	0.6	**Total**	**623**	**100**

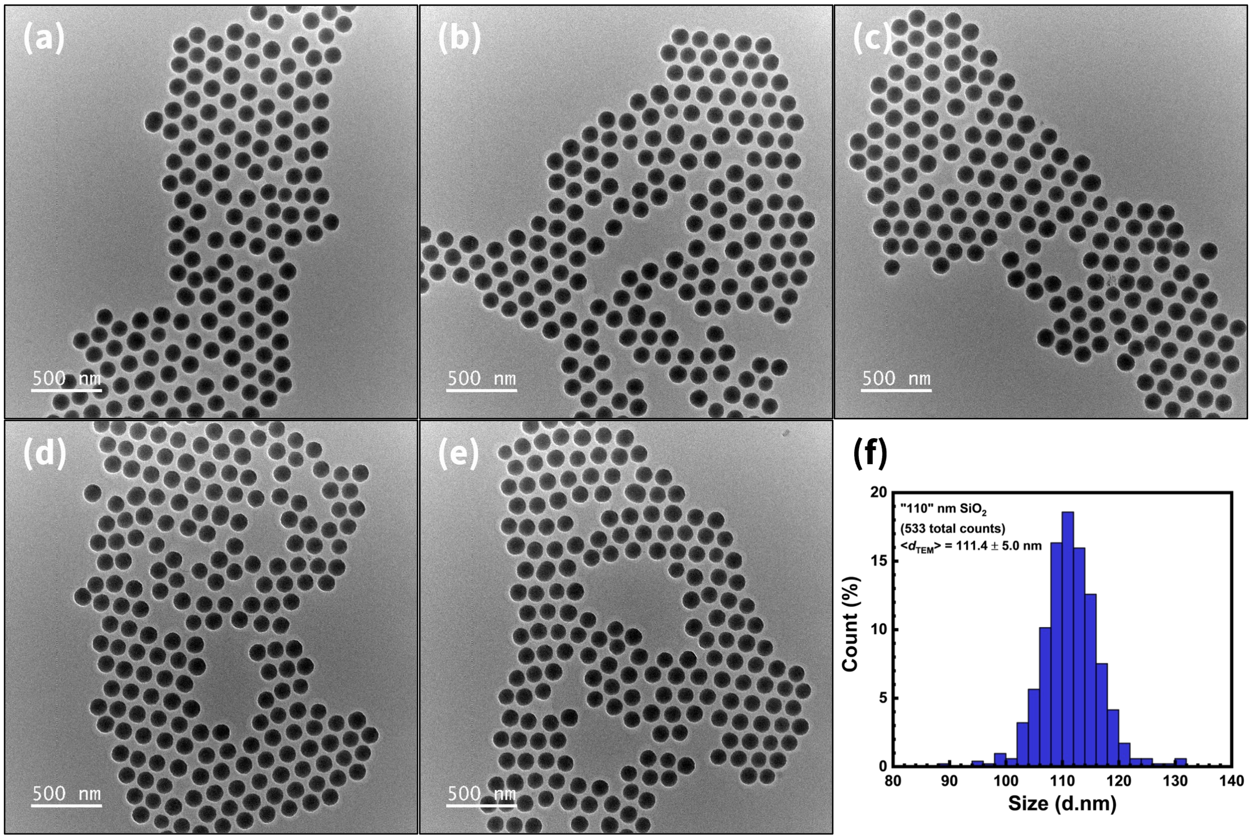

Figure S4. (a-e) Representative TEM images of SiO_2-*g*-PMMA (after model reactions) with average NP diameters of around 110 nm using a random sampling method. (f) Diameter distributions with calculated average of d_{large} = 111.4 ± 5.0 nm.

Table S2. Core Diameter Distributions of SiO_2 NPs (533 total counts, *d* ~ 110 nm).

Size (nm)	Counts	Count (%)	Size (nm)	Counts	Count (%)	Size (nm)	Counts	Count (%)
88-90	1	0.2	104-106	30	5.6	120-122	9	1.7
90-92	0	0.0	106-108	54	10.1	122-124	3	0.6
92-94	0	0.0	108-110	87	16.3	124-126	3	0.6
94-96	2	0.4	110-112	99	18.6	126-128	1	0.2
96-98	1	0.2	112-114	85	15.9	128-130	1	0.2
98-100	5	0.9	114-116	67	12.6	130-132	3	0.6
100-102	3	0.6	116-118	40	7.5			
102-104	17	3.2	118-120	22	4.1	**Total**	**533**	**100**

S3. PMMA Grafting from 15 nm SiO_2-Br with Varied Initiator Density.

Monomer conversion estimated from gravimetry.

Two aluminum pans were pre-weighed, and their masses recorded as m_{pan}. Before purging nitrogen (marked as t_0), a small aliquot of the mixture was withdrawn and loaded into one pre-weighed pan. The combined mass was recorded as m_{total}. The loaded pan was placed in an oven at 100 °C for 12 h to remove anisole, assumed to evaporate completely under these conditions. The dried sample with the pan was weighed as m_{dry}. At the end of the polymerization, the same procedure was repeated with the second pre-weighed pan (marked as t_p).

Detailed calculation procedures are as follows.

(i) Mass before drying:

$$\mathrm{m}_{<\mathrm{oven}} = \mathrm{m}_{\mathrm{total}} - \mathrm{m}_{\mathrm{pan}} \quad \text{(S5)}$$

(ii) Mass after drying:

$$\mathrm{m}_{\mathrm{oven}>} = \mathrm{m}_{\mathrm{dry}} - \mathrm{m}_{\mathrm{pan}} \quad \text{(S6)}$$

(iii) Mass evaporated:

$$\mathrm{m}_{\mathrm{evap}} = \mathrm{m}_{<\mathrm{oven}} - \mathrm{m}_{\mathrm{oven}>} \quad \text{(S7)}$$

(iv) Non-evaporated fraction ratio:

$$f_{\mathrm{non-evap}} = \frac{\mathrm{m}_{\mathrm{oven}>}}{\mathrm{m}_{<\mathrm{oven}}} \quad \text{(S8)}$$

(v) Non-evaporated mass:

$$\mathrm{m_{non-evap}} = f_{\mathrm{non-evap,\ t_0}} \times \mathrm{m_{<oven}} \quad \text{(S9)}$$

(vi) Combined mass of MMA, PMMA, and anisole:

$$\mathrm{m_{com}} = \mathrm{m_{<oven}} - \mathrm{m_{non-evap}} \quad \text{(S10)}$$

(vii) PMMA mass:

$$\mathrm{m_{PMMA}} = \mathrm{m_{com}} - \mathrm{m_{evap}} \quad \text{(S11)}$$

(viii) Given that MMA (ρ_{MMA} = 940 kg m^{-3}) is 50 vol% in anisole (ρ_{anisole} = 995 kg m^{-3}), anisole mass:

$$\mathrm{m_{anisole}} = \mathrm{m_{com}} \times \frac{995}{995 + 940} \quad \text{(S12)}$$

(ix) The unreacted MMA mass:

$$\mathrm{m_{MMA}} = \mathrm{m_{com}} - \mathrm{m_{PMMA}} - \mathrm{m_{anisole}} \quad \text{(S13)}$$

(x) The ratio of unreacted MMA to anisole:

$$F_{\mathrm{MMA/anisole}} = \frac{\mathrm{m_{MMA}}}{\mathrm{m_{anisole}}} \quad \text{(S14)}$$

(xi) Monomer conversion ($\mathrm{conv_1}$):

$$\mathrm{conv_1} = 1 - \frac{F_{\mathrm{MMA/anisole,\ t_p}}}{F_{\mathrm{MMA/anisole,\ t_0}}} \quad \text{(S15)}$$

Monomer conversion estimated from inorganic fraction.

The weight of grafted PMMA can be estimated based on the mass of the SiO_2-Br macroinitiator and the inorganic fraction of the resulting SiO_2-*g*-PMMA:

$$\mathrm{m_{PMMA,grafted}} = \frac{\mathrm{m_{SiO_2-Br}}}{f_{\mathrm{inorg}}} - \mathrm{m_{SiO_2-Br}} \tag{S16}$$

Accordingly, the monomer conversion derived from the inorganic fraction ($\mathrm{conv_2}$) can be expressed as:

$$\mathrm{conv_2} = \frac{\mathrm{m_{PMMA,grafted}}}{\mathrm{m_{monomer}}} \tag{S17}$$

Table S3. Monomer Conversions Estimated from Gravimetry and Inorganic Fraction for PMMA-Grafted SiO_2 Nanoparticles Prepared via SI-ARGET ATRP.

Entry			$conv_1$ [a]	m_{silica}	f_{inorg} [b]	$conv_2$ [c]	Dev [d]
d (nm)	σ_{in} (Br nm^{-2})	$[Cu^{II}Br_2]_0$ (ppm)	(%)	(mg)	(%)	(%)	(%)
15	0.7	100	3.2	28.1	21	3.9	− 0.6
15	0.7	30	7.6	28.1	10	8.6	− 1.1
15	0.7	10	5.5	28.1	12	7.5	− 2.0
15	0.7	3	4.5	28.1	16	5.2	− 0.6
15	0.7	1	5.4	28.1	17	4.9	+ 0.4
15	0.7	0.3	3.3	28.1	24	3.2	+ 0.1
15	0.4	100	4.2	49.1	27	4.7	− 0.5
15	0.4	30	7.5	49.1	17	8.5	− 1.0
15	0.4	10	7.9	49.1	14	10.3	− 2.4
15	0.4	3	4.1	49.1	26	4.9	− 0.7
15	0.4	1	3.6	49.1	31	3.9	− 0.2
15	0.4	0.3	3.6	49.1	30	4.0	− 0.4
15	0.1	100	7.5	196.7	44	8.9	− 1.4
15	0.1	30	6.3	196.7	54	5.9	+ 0.4
15	0.1	10	6.5	196.7	47	7.8	− 1.2
15	0.1	3	6.3	196.7	83	1.4	+ 5.0
15	0.1	1	0.9	196.7	77	2.1	− 1.3
15	0.1	0.3	0.7	196.7	78	2.0	− 1.3
15	0.03	100	/	655.8	70	10.1	/
15	0.03	30	/	655.8	79	6.4	/
15	0.03	10	/	655.8	87	3.5	/
15	0.03	3	/	655.8	94	1.5	/
15	0.03	1	/	655.8	91	2.3	/
15	0.03	0.3	/	655.8	84	4.5	/
75	0.7	100	4.7	75.2	40	4.1	+ 0.6
75	0.7	30	6.9	75.2	30	6.4	+ 0.5
75	0.7	10	4.7	75.2	37	4.5	+ 0.3
75	0.7	3	5.1	75.2	40	4.0	+ 1.1
75	0.7	1	4.9	75.2	49	2.7	+ 2.2

**75**	**0.7**	**0.3**	2.0	75.2	79	0.7	+ 1.3
**75**	**0.5**	**100**	4.7	105.3	48	4.1	+ 0.6
**75**	**0.5**	**30**	5.7	105.3	43	5.1	+ 0.6
**75**	**0.5**	**10**	3.5	105.3	56	3.0	+ 0.5
**75**	**0.5**	**3**	4.8	105.3	51	3.6	+ 1.2
**75**	**0.5**	**1**	5.7	105.3	52	3.4	+ 2.3
**75**	**0.5**	**0.3**	3.2	105.3	68	1.8	+ 1.5
**110**	**0.7**	**100**	4.6	109.6	49	4.0	+ 0.6
**110**	**0.7**	**30**	2.5	109.6	62	2.4	+ 0.1
**110**	**0.7**	**10**	2.8	109.6	58	2.8	+ 0.0
**110**	**0.7**	**3**	2.4	109.6	65	2.1	+ 0.2
**110**	**0.7**	**1**	2.8	109.6	60	2.5	+ 0.3
**110**	**0.7**	**0.3**	5.0	109.6	72	1.5	+ 3.5
**110**	**0.5**	**100**	6.1	153.4	49	5.6	+ 0.4
**110**	**0.5**	**30**	7.4	153.4	44	6.9	+ 0.4
**110**	**0.5**	**10**	6.3	153.4	51	5.3	+ 0.9
**110**	**0.5**	**3**	4.8	153.4	58	4.0	+ 0.8
**110**	**0.5**	**1**	6.5	153.4	59	3.8	+ 2.7
**110**	**0.5**	**0.3**	2.9	153.4	77	1.6	+ 1.3

[a] Monomer conversion 1 was estimated gravimetrically (eqs S5-S15). Values were not reported for SiO_2-Br with d = 15 nm, σ_{in} = 0.03 Br nm^{-2} due to incomplete dissolution. [b] Inorganic weight fraction determined by TGA under air. [c] Monomer conversion 2 was estimated from the inorganic fraction (eqs S16-S17) and is considered more reliable. [d] Monomer conversion deviation was calculated as $conv_1 - conv_2$.

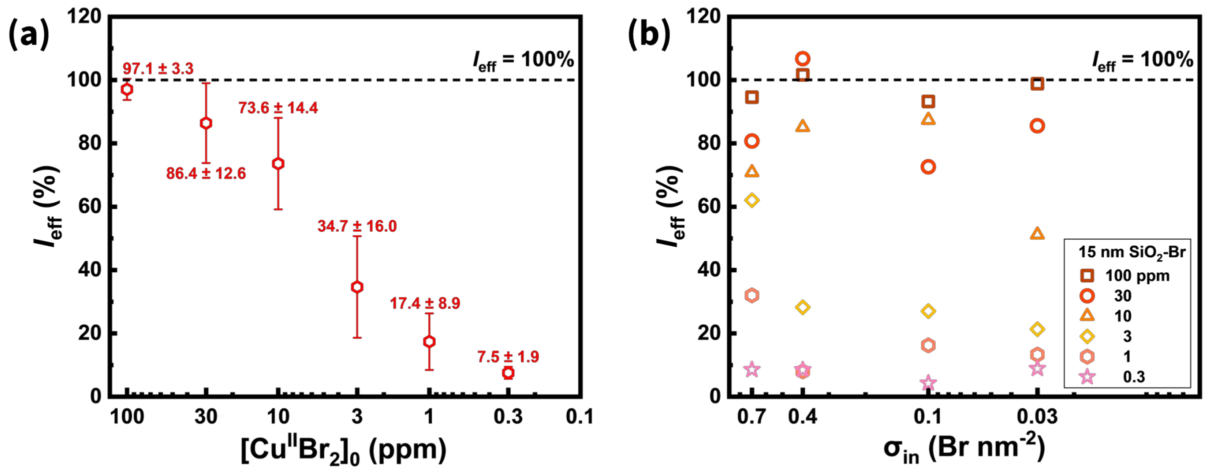

Figure S5. SiO_2-*g*-PMMA (d_{small} ~ 15 nm) via SI-ARGET ATRP. (a) Average Initiation efficiency (I_{eff}) values with standard deviations calculated across samples prepared at the same initial Cu catalyst concentrations ([Cu^{II}Br$_2$]$_0$). (b) I_{eff} as the function of initiator density (σ_{in} in the logarithmic scale) at varied [Cu^{II}Br$_2$]$_0$.

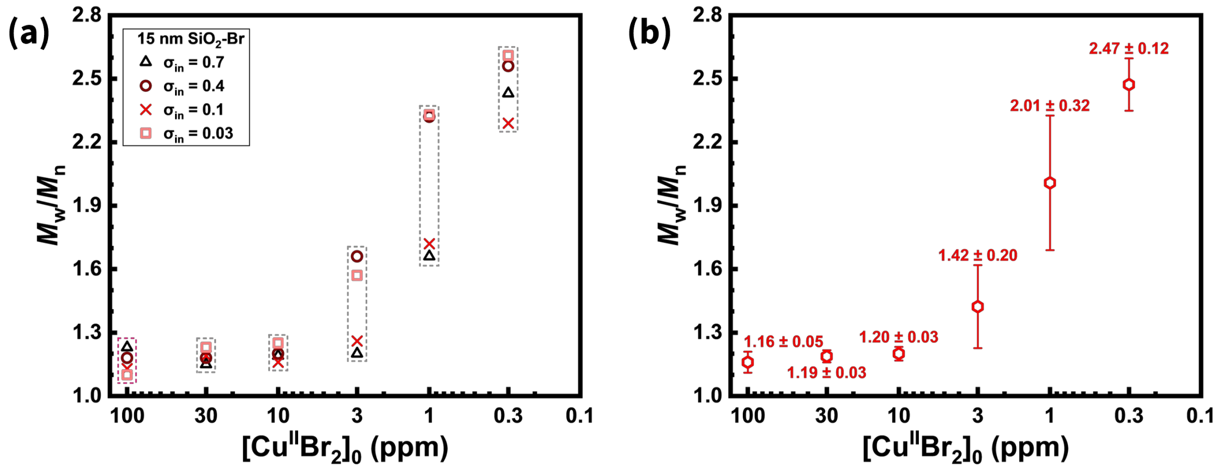

Figure S6. Molecular weight distribution (M_w/M_n) of the grafted PMMA layers from SiO_2-Br nanoparticles (d_{small} ~ 15 nm) via SI-ARGET ATRP at varied initiator density (σ_{in}) and initial Cu catalyst concentrations ([Cu^{II}Br$_2$]$_0$). (a) Individual data points for all samples (as Figure 4a). (b) Average M_w/M_n values with standard deviations calculated across samples prepared at the same [Cu^{II}Br$_2$]$_0$.

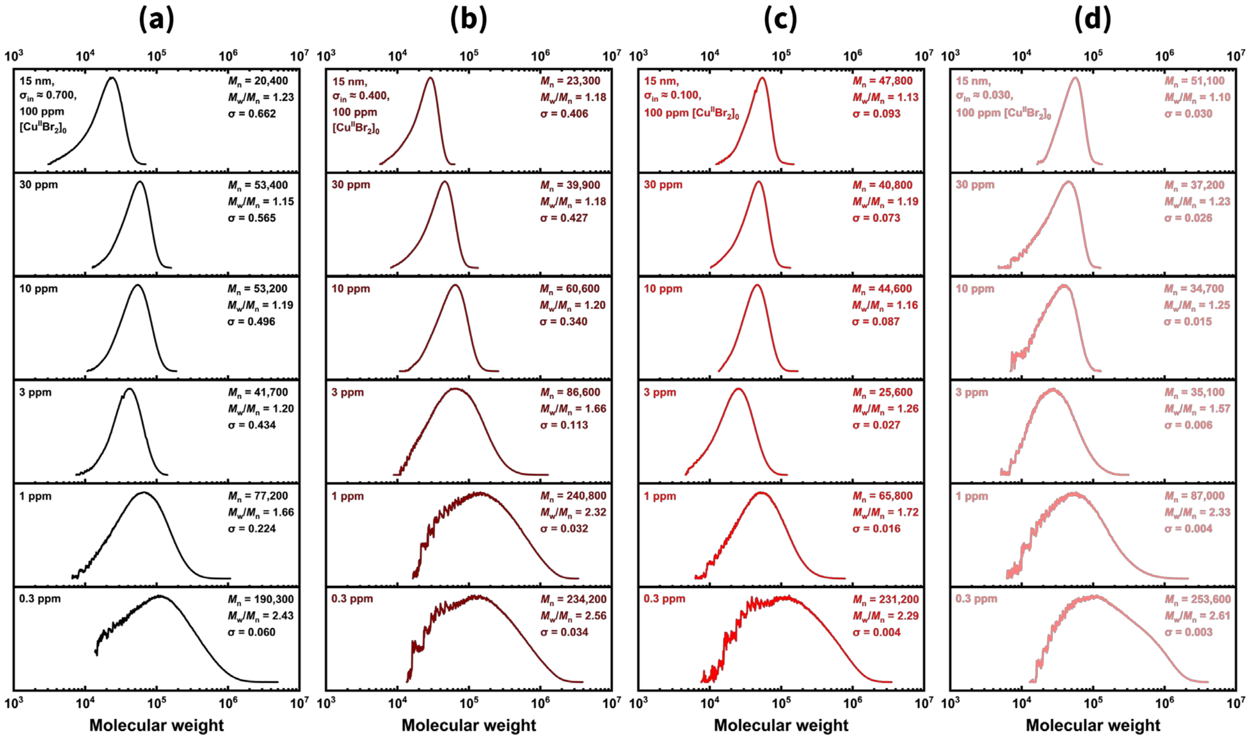

Figure S7. Normalized GPC analysis of number-average molecular weight (M_n) distribution for 15 nm SiO_2-*g*-PMMA with σ_{in} = (a) 0.7, (b) 0.4, (c) 0.1, and (d) 0.03 Br nm^{-2}. In each panel (from top to bottom), $[Cu^{II}Br_2]_0$ was systematically decreased (100/30/10/3/1/0.3 ppm relative to monomer).

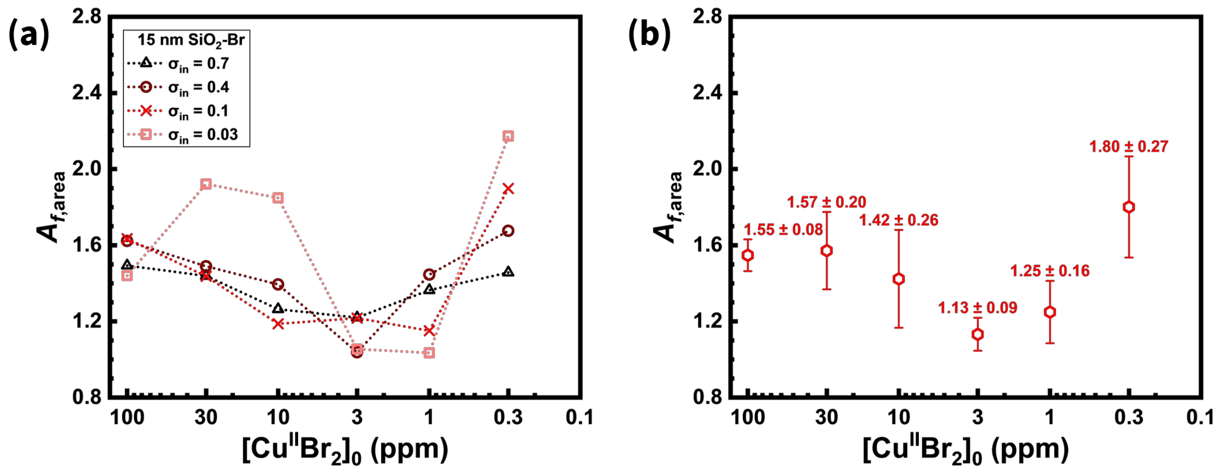

Figure S8. Asymmetry factor of area ($A_{f,\mathrm{area}}$) of PMMA brushes grafting from SiO_2-Br nanoparticles (d_{small} ~ 15 nm) via SI-ARGET ATRP at varied initiator density (σ_{in}) and initial Cu catalyst concentrations ($[Cu^{II}Br_2]_0$). (a) Individual data points for all samples (as Figure 4c). (b) Average $A_{f,\mathrm{area}}$ values with standard deviations calculated across samples prepared at the same $[Cu^{II}Br_2]_0$.

S4. PMMA Grafting from 75 and 110 nm SiO_2-Br with Varied Initiator Density.

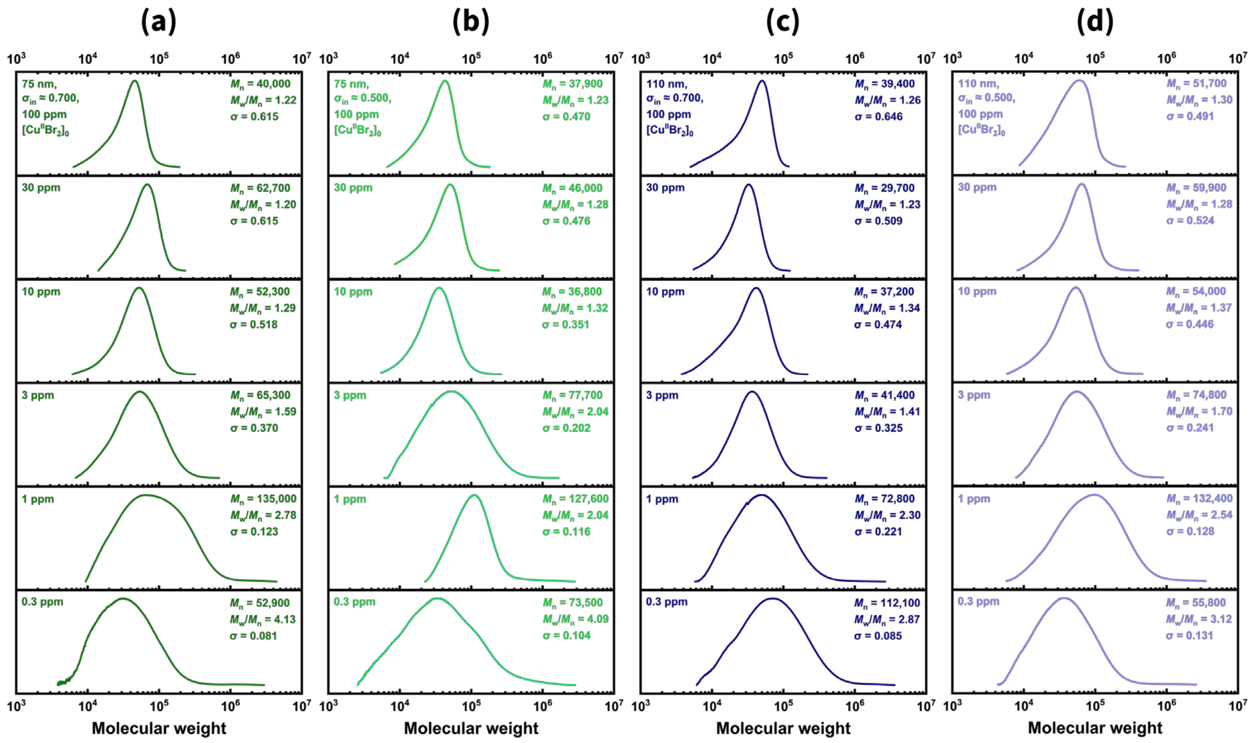

Figure S9. Normalized GPC analysis of number-average molecular weight (M_n) distribution for SiO_2-*g*-PMMA with size and σ_{in} of (a) 75 nm, σ_{in} = 0.7 Br nm^{-2}, (b) 75 nm, σ_{in} = 0.5 Br nm^{-2}, (c) 110 nm, σ_{in} = 0.7 Br nm^{-2}, and (d) 110 nm, σ_{in} = 0.5 Br nm^{-2}. In each panel (from top to bottom), $[Cu^{II}Br_2]_0$ was systematically decreased (100/30/10/3/1/0.3 ppm relative to monomer).

S5. GPC Analysis of Unattached PMMA.

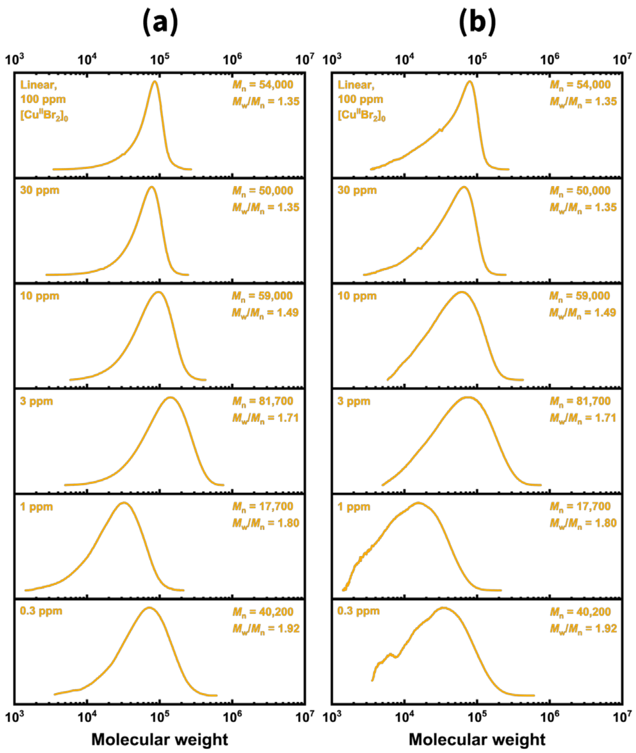

Figure S10. Normalized GPC analysis of (a) weight- and (b) number-average molecular weight distribution of unattached PMMA. From top to bottom, $[Cu^{II}Br_2]_0$ was systematically decreased (100/30/10/3/1/0.3 ppm relative to monomer).
